# Synthesis and Anticancer Activity of 3,4-Diaryl-1,2-dihydro- and 1,2,3,4-Tetrahydroquinolines

**DOI:** 10.3390/molecules29174273

**Published:** 2024-09-09

**Authors:** Santosh Rajput, Valerio Falasca, Mohan Bhadbhade, David StC Black, Naresh Kumar

**Affiliations:** 1 School of Chemistry, University of New South Wales, Sydney, NSW 2052, Australia; 2 Solid State and Elemental Analysis Unit, Mark Wainwright Analytical Centre, The University of New South Wales, Sydney, NSW 2052, Australia

**Keywords:** tetrahydroquinolines, dihydroquinolin-4-ones, quinolones, Grignard reactions, dehydration, hydrogenation

## Abstract

Tetrahydroquinolines are key structures in a variety of natural products with diverse pharmacological utilities and other applications. A series of 3,4-diaryl-5,7-dimethoxy-1,2,3,4-tetrahydroquinolines were synthesized in good yield by reacting 3-aryl-5,7-dimethoxy-2,3-dihydroquinolin-4-ones with different Grignard reagents followed by the dehydration of the intermediate phenolic compounds. Subsequent reduction and deprotection were carried out to achieve the desired tetrahydroquinolone moiety. The lead compound **3c** showed low micromolar inhibition of various cancer cell lines. Demethylation under different reaction conditions was also investigated to afford the corresponding monohydroxy analogues.

## 1. Introduction

Quinolones and their derivatives occur in numerous natural products, and many of them display interesting biological activities. In particular, hydrogenated quinoline moieties are important structures in various natural products that exhibit a broad range of biological properties and potential pharmaceutical applications [1].

Rapid development in the chemistry of tetrahydroquinolines has been observed in recent years, because they are core structures in many important pharmacological agents [2] and drug molecules, such as antiarrhythmic and cardiovascular agents, anticancer drugs, and immunosuppressants, and ligands for 5-HTTA and NMDA receptors [3]. Besides their pharmaceutical applications, tetrahydroquinoline derivatives are useful as pesticides [4,5], antioxidants [6,7,8], and corrosion inhibitors [9] and are active components of various dyes [10,11]. In addition, they also have found application in modern recording technologies [12,13].

Many relatively simple 1,2,3,4-tetrahydroquinolines have been tested as potential drugs. Amongst them are oxamniquine, a schistosomicide [14,15,16], virantmycin, a novel antibiotic [17,18,19], and nicainoprol, an antiarrhythmic drug [20,21,22]. Tetrahydroquinolin L-689560 is also one of the most potent NMDA antagonists yet found [23,24,25].

Several compounds of this class are found to occur in nature. For example, discorhabdin C, a polycyclic system based on tetrahydroquinoline, is a marine alkaloid [2,26,27] and dynemicin A, a natural antitumor antibiotic, has a complex structure built on the tetrahydroquinoline nucleus [28,29]. A 2,4,6-trisubstituted tetrahydroquinoline, isolated from Martinella iquitosensis, exhibits activity as a bradykinin antagonist against α-adrenergic and histaminergic receptors [30].

2-Substituted tetrahydroquinolines have also recently been discovered as a class of natural products, that includes angustureine, cuspareine, oxamniquine, and galipeine. Some members of this family exhibit interesting pharmacological properties [31].

The dihydroquinolone structure can be found in a variety of natural products [32,33] and in a large number of compounds, which display biological activity. For example, 2,2,4-trisubstituted-1,2-dihydroquinolines have been used to produce potent compounds that possess antibacterial, antidiabetic, and anti-inflammatory activities [34]. Compounds possessing this motif have also been shown to act as lipid peroxidation inhibitors, 4 HMG-CoA reductase inhibitors, ileal bile acid transporter inhibitors, and progesterone agonists and antagonists [34].

**Figure 1:**
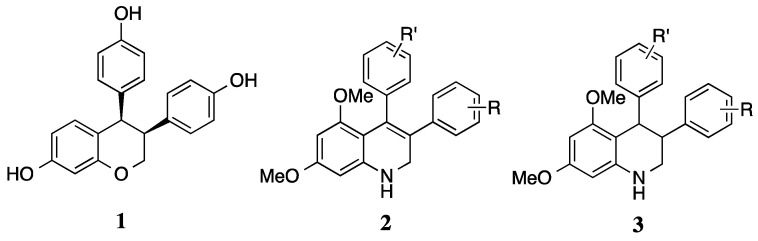
Structures of isoflavone **1** and target compounds **2** and **3**.

Due to the diverse biological activities exhibited by this class of compounds, we were interested in synthesizing dihydrogenated and tetrahydrogenated derivatives of quinolines. It was envisaged that these hydrogenated compounds would show promising biological activities, due to the pharmacological applications shown by the isoflavone class of compounds. The isoflavone analogue triphendiol **1** (Figure 1) has recently been granted orphan drug status by the FDA for pancreatic and bile duct cancers and late-stage melanoma. Inspired by the success of triphendiol **1** it was decided to introduce an aryl group at the C4 position of the 3-substituted quinolone ring system and keep the oxygenation pattern similar to that of natural isoflavones to generate azaisoflavene **2** and azaisoflavan **3** structures. Furthermore, many biologically active isoflavones suffer from poor oral bioavailability and rapid CYP450 metabolism [35,36]. Our objective in this study was to synthesize biologically active tetrahydroquinolines with improved solubility and metabolic stability compared to current isoflavone analogues. The biological activity of the tetrahydroquinolines was investigated through a single concentration screen in different cancer cell lines.

## 2. Results and Discussion

The project mainly involved the synthesis of a series of 3,4-diaryl-5,7-dimethoxy 1,2-dihydro and 1,2,3,4-tetrahydroquinolines. Various demethylating conditions were investigated in order to obtain the corresponding hydroxyl analogues, which could possibly show greater biological activity. 

### 2.1. Attempted Synthesis of 3,4-Diaryl-1,2-dihydroquinolines by Nucleophilic Addition

It was envisaged that 3,4-diarylazaisoflavenes **2** could be obtained by the nucleophilic addition of aryl groups to the carbonyl group of 2,3-dihydroquinolin-4-ones to give the corresponding 3,4-diarylazaisoflavan-4-ols, which subsequently could undergo dehydration to give the desired target compounds.

For this, 3-substituted-5,7-dimethoxy-2,3-dihydroquinolin-4(1*H*)-one **4** was treated with arylmagnesium bromide in THF at reflux for 4 h to give 3,3-diaryl-5,7-dimethoxy-1,2,3,4-tetrahydroquinolin-4-ols **5** in 72–77% yield. The intermediate **5a** was characterized by its ^1^H NMR spectrum, which showed two doublets of doublets at δ 3.12 and 3.24 ppm (*J* = 3.0, 9.0 Hz) corresponding to the two H2 protons. The H3 proton appeared as a multiplet at δ 3.46 ppm. The OH group was seen as a singlet at δ 3.79 ppm and the phenyl ring protons appeared as a multiplet at δ 7.11–7.20 ppm.

**Scheme 1:**
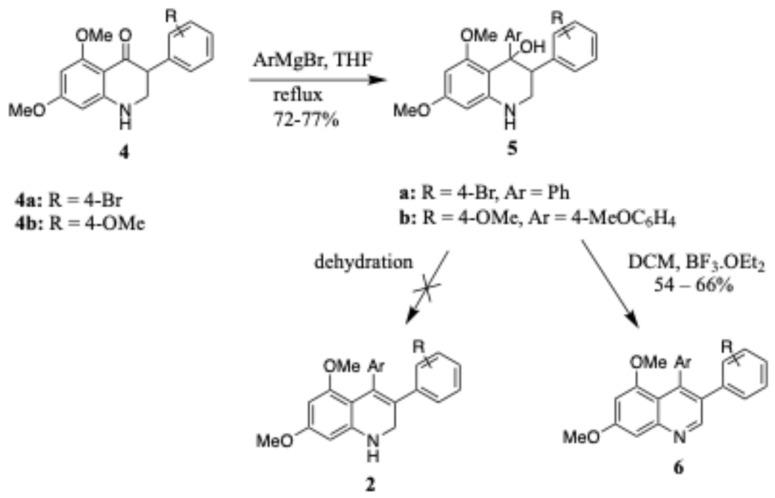
Grignard reaction on 2,3-dihydroquinolin-4-ones.

The acid-catalyzed dehydration of **5** did not result in the formation of the desired 3,4-diaryl-1,2-dihydroquinoline **2** but was found to preferentially generate the more highly stabilized 3,4-diaryl-5,7-dimethoxyquinoline **6** in 54–66% yield instead (Scheme 1). The formation of the quinoline moiety can be attributed to further dehydrogenation presumably due to aerial oxidation, occurring together with the dehydration reaction. The quinoline structure was confirmed by ^1^H NMR spectroscopy, which showed the disappearance of the aliphatic protons H2 and H3 present in **5**. The presence of a significantly downfield shifted singlet at δ 8.75 ppm in **6** correlating to H2 was the key resonance signal for the identification of compound **6**. 

This result was found to be consistent with literature reports [37,38], which state that 1,2-dihydroquinolines that are unsubstituted at the nitrogen atom and have at least one hydrogen at C2 are unstable. These dihydroquinolines are rapidly oxidized in air to the quinoline or undergo disproportionation by trace acids to give a mixture of the quinoline and tetrahydroquinoline. 

To overcome this problem, it was decided to protect the NH group and then perform the Grignard reaction on the *N*-protected dihydroquinolin-4-ones to give the corresponding *N*-protected azaisoflavan-4-ol, followed by dehydration and deprotection to give the desired 3,4-diaryl-azaisoflavenes **2**.

Hence, compound **7** was heated at reflux for 4 h with aryl magnesium bromide. This reaction gave a mixture of 3,4-diaryl-5,7-dimethoxy-1,2,3,4-tetrahydroquinolin-4-ol **5** and ethyl-3,4-diaryl-4-hydroxy-5,7-dimethoxy-3,4-dihydroquinoline-1(2*H*)-carboxylate **8**. The ratio of **5** to **8** was found to be dependent upon the reaction conditions. The use of three equivalents of arylmagnesium bromide afforded **5** and **8** in 30% and 50% yield, respectively, whereas the use of two equivalents of arylmagnesium bromide gave only **8** in 78–88% yield. The structure of compound **8** was confirmed by ^1^H NMR spectroscopy data. Compound **8a** showed the presence of the COOEt group as a triplet and a quartet at δ 1.19 ppm (*J* = 6.0 Hz) and 4.15 ppm (*J* = 6.0 Hz) for the CH_3_ and CH_2_ groups, respectively. The OH proton was present as a singlet at δ 5.04 ppm and the phenyl ring protons as a multiplet at δ 7.07–7.14 ppm. The ^1^H NMR data indicate that **8** was formed as a single diastereomer. Due to the presence of an aryl substituent at the C3 position, we assume that the Grignard reagent attacks the planar carbonyl group from the opposite side to the aromatic ring.

The acid-catalyzed dehydration of **8** with BF_3_.OEt_2_ in DCM resulted in the formation of ethyl 3,4-diaryl-5,7-dimethoxy-quinoline-1(2*H*)-carboxylate **9** in 52–58% yield. The structure of compound **9** was also confirmed by ^1^H NMR spectroscopy data. Compound **9a** indicated the disappearance of the H3 proton and the appearance of a singlet at δ 4.54 ppm integrating for two protons, correlating to H2 and a triplet at δ 1.29 ppm (*J* = 9.0 Hz) and quartet at δ 4.24 ppm (*J* = 9.0 Hz) indicating the presence of the protecting group, -COOEt.

**Scheme 2:**
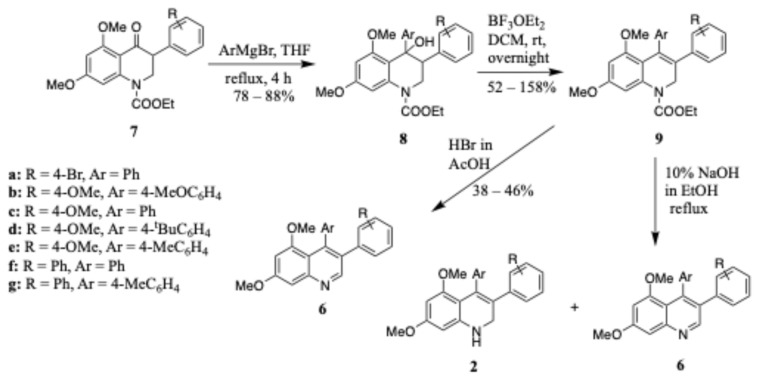
Grignard reaction on *N*-protected 2,3-dihydroquinolin-4-ones.

Subsequent deprotection of **9** was investigated using both acidic and basic conditions under an inert atmosphere. When HBr in AcOH [39] was utilized, **9** underwent dehydrogenation in addition to deprotection, giving the more stable quinoline moiety **6**. Alternatively, heating **9** at reflux with 10% NaOH in EtOH for 5 h generated a mixture of two compounds by TLC (Scheme 2). Separation of these two compounds was attempted using various methods, such as column chromatography, crystallization, and preparative TLC, but was ultimately unsuccessful. When the crude mixture was analyzed using ^1^H NMR spectroscopy, it was found to contain compounds **2** and **6** in an approximate ratio of 30:70. The presence of compound **2** was confirmed by the DEPT-135 NMR experiment, which showed the presence of a CH_2_ at δ 47.5 ppm.

Therefore, the proposed synthesis of 3,4-diaryl-5,7-dimethoxy-1,2-dihydroquinoline **2** could not be accomplished via the methodologies used in this project. Instead, the more stable quinoline derivatives **6** were prepared in similar yields using the two strategies outlined in Scheme 1 and Scheme 2. 

### 2.2. Synthesis of 3,4-Diaryl-1,2,3,4-tetrahydroquinolines

The literature indicates that direct reduction of quinolines is the most efficient method of preparing tetrahydroquinolines [40]. Direct reduction of the 2,3-dihydroquinolin-4-ones is also possible [41,42,43], but this would lead to a C4-unsubstituted product. Hence, attempts were made to reduce the quinoline **6** to 3,4-diaryl-tetrahydroquinoline **3** directly by applying two different reduction conditions. The first one involved heating the quinoline **6** with LAH at reflux and the second involved catalytic hydrogenation with hydrogen over palladium on charcoal. However, both reaction conditions gave multiple spots by TLC, which could not be separated to allow successful identification of the compounds formed.

In another approach, it was decided to reduce the *N*-substituted 3,4-diarylazaisoflavenes **9** to *N*-substituted 3,4-diaryl-tetrahydroquinolines **10** and then investigate the deprotection of **10** to synthesize the 3,4-diaryl tetrahydroquinoline **3**.

**Scheme 3:**
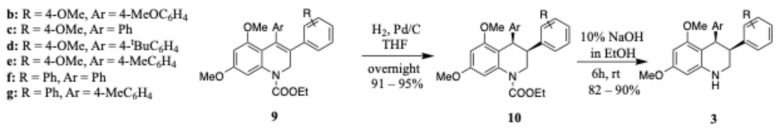
Synthesis of 3,4-diaryl-1,2,3,4-tetrahydroquinolines.

For instance, ethyl-3,4-diaryl-5,7-dimethoxy-quinoline-1(2*H*)-carboxylate **9** was hydrogenated in THF using Pd/C and hydrogen gas overnight to give ethyl-3,4-diaryl-5,7-dimethoxy-3,4-dihydroquinoline-1(2*H*)-carboxylate **10** in 91–95% yield (Scheme 3). The structure of compound **10** was confirmed by ^1^H NMR spectroscopy data. Compound **10** showed a doublet of a triplet for the H3 proton at δ 3.36 ppm (*J* = 3.0, 12.0 Hz) and a doublet for the H4 proton at δ 4.45 ppm (*J* = 3.0 Hz) and triplet at δ 3.67 ppm (*J* = 12.0 Hz) and a doublet of doublet of doublet at δ 4.09 ppm (*J* = 3.0, 6.0, 12.0 Hz) for two H2 protons. 

**Figure 2:**
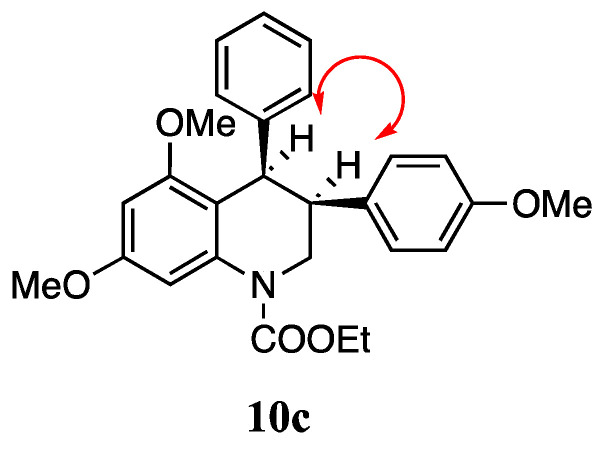
NOE correlations of compound **10c**.

The expected *cis* stereochemistry of the compound 10 was established on the basis of the coupling constant of *J* = 3.0 between H3 and H4. Moreover, NOE correlations between the H3 and H4 protons were evident in the 2D NMR experiment, further confirming the *cis* configuration (Figure 2 and Figure S11).

**Figure 3:**
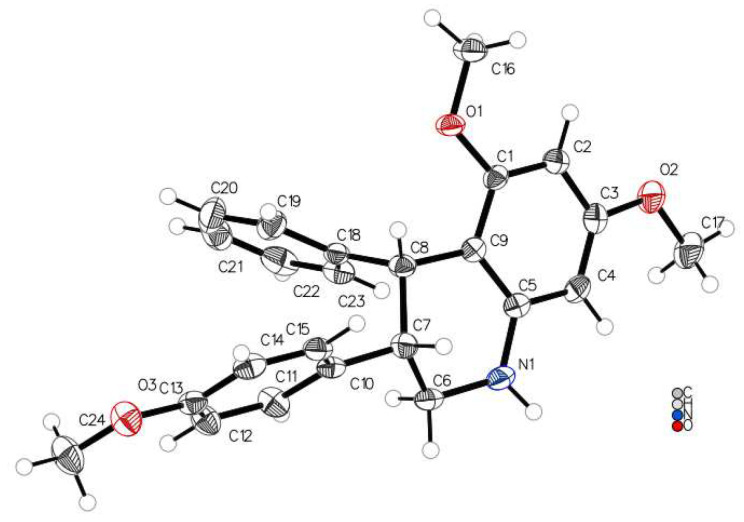
ORTEP diagram of compound **3c**.

The deprotection of compound **10** was carried out by heating at reflux in 10% NaOH in EtOH for 6 h to give the desired 5,7-dimethoxy-3,4-diaryl-1,2,3,4-tetrahydroquinolines **3** in 82–90% yield. The structure of **3** was confirmed by the lack of protons correlating to the –COOEt group in the ^1^H NMR spectrum and the presence of a NH proton as a singlet at δ 4.14 ppm. The coupling constant between H3 and H4 protons (*J* = 3.0 Hz) indicated the *cis* configuration had been retained, and this was further confirmed by X-ray crystallographic analysis. The ORTEP diagram of compound **3c** is shown in Figure 3. Packing of molecules was dominated by CH_3_–π and slipped π–π interactions (Figure S1).

Using this approach, some 3,4-diaryl-5,7-dimethoxytetrahydroquinoline analogues (Table 1) were synthesized and their spectroscopic data were found to be consistent with those of compounds **3c**.

### 2.3. Demethylation of 3,4-Diaryl-5,7-dimethoxy-1,2,3,4-tetrahydroquinolines

After synthesizing the dimethoxy-substituted tetrahydroquinolines, the subsequent aim was to investigate suitable demethylation conditions to generate the hydroxyl analogues.

**Scheme 4:**
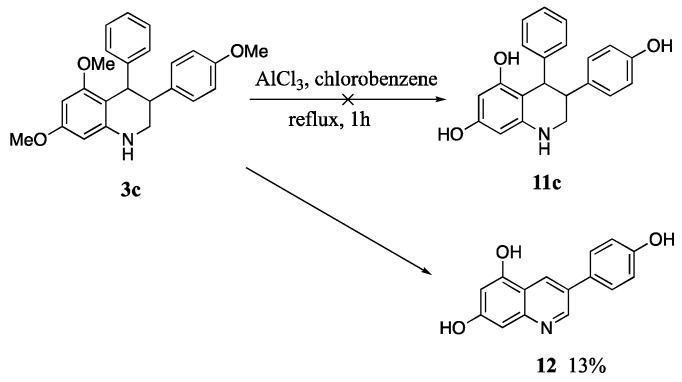
Demethylation using AlCl_3_ in chlorobenzene.

Initially, tetrahydroquinoline **3c** was heated at reflux with aluminum chloride in chlorobenzene for 1 h. Two compounds were obtained after aqueous work up and column chromatography. However, only one compound was pure enough to be characterized as **12** in 13% yield (Scheme 4). The reaction with AlCl_3_ therefore resulted in subsequent aromatization and dearylation in addition to demethylation.

The second compound that was obtained from this reaction is hypothesized to be **11c**, however, its purity was very poor and decomposed during purification. Meanwhile, the ^1^H NMR spectrum of compound **12** lacked the phenyl group protons at C4 in addition to the methoxy protons. Three signals as a singlet corresponding to the hydroxyl protons were present at δ 9.40, 9.69, and 10.26 ppm, which confirmed that the demethylation had occurred. Doublets at δ 8.19 ppm and 8.75 ppm (*J* = 2.4 Hz) for H3 and H4 indicated that aromatization had occurred. The structure of compound **12** was further confirmed by HRMS, giving a molecular ion peak [M + H]^+^ at 254.0805 corresponding to the molecular formula. 

**Scheme 5:**
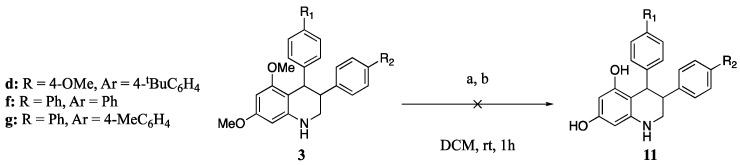
Demethylation of **3**: (a) = 7 equiv. BBr_3_; (b) **3f** = 5 equiv. BBr_3_.

To improve the yield and the purity of the dihydroxy analogues, BBr_3_ in DCM was examined as an alternate demethylating agent. According to the previous results, two equivalents of BBr_3_ were required for the cleavage of one methoxy group. Hence, the same strategy was applied and tetrahydroquinoline **3e** was stirred with seven equivalents of BBr_3_ in DCM at room temperature for 1 h (Scheme 5). However, the product of this reaction could not be purified even after several attempts. This could possibly be due to the presence of three hydroxyl groups in compound **11** rendering the compound significantly unstable. Therefore, it was decided to demethylate tetrahydroquinoline **3f**, which has only two methoxy groups, with five equivalents of BBr_3_ in DCM at room temperature for 1 h (Scheme 5). However, purification of compound **11f** proved to be similarly problematic and pure spectra of the product could not be obtained.

**Scheme 6:**
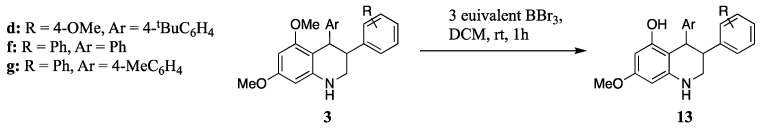
Demethylation of **3f** using BBr_3_ (3 equiv.) in DCM.

In an attempt to improve the purity of the hydroxyl compounds, the number of equivalents of BBr_3_ was reduced. Tetrahydroquinoline **3f** was stirred with three equivalents of BBr_3_ in DCM for 1 h. Work up and purification with column chromatography resulted in a 38% yield of compound **13f** (Scheme 6, Table 2). The ^1^H NMR spectrum of compound **13f** still showed the presence of a methoxy group at δ 3.69 ppm and only one hydroxyl group was evident at δ 7.78 ppm. To confirm the structure of this compound a NOE experiment was performed, which showed a correlation between the methoxy and the H6 and H8 protons while the hydroxy signal only correlated to the H6 proton. This indicated that demethylation had occurred at the C5 position, thus giving 5-hydroxy-7-methoxytetrahydroquinoline **13f**. 

By the application of this strategy three monohydroxyl analogues were synthesized (Table 2).

### 2.4. Biological Activity of 3,4-Diaryl-1,2,3,4-tetrahydroquinolines

**Figure 4:**
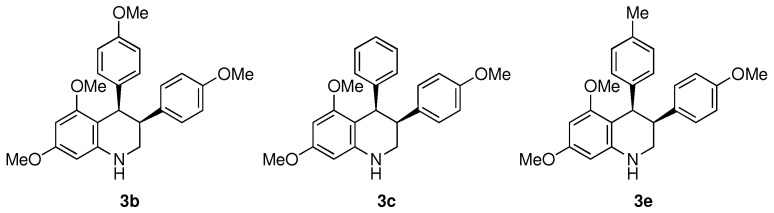
Lead anticancer screen compounds.

A selection of the synthesized methoxy tetrahydroquinoline compounds were screened for their anticancer activity (Figure 4).

The compounds selected for this screen gave a valuable insight into the structure–activity relationship of functionality of both aryl groups of the tetrahydroquinolines (Figure 4, Table S1). In vitro growth inhibition assays were performed at a fixed concentration of 25 μM in a range of cancer cell lines, including H460 lung carcinoma, DU145 prostate carcinoma, A-431 skin (epidermoid) carcinoma, HT-29 colon adenocarcinoma, and MCF7 breast adenocarcinoma.

**Figure 5:**
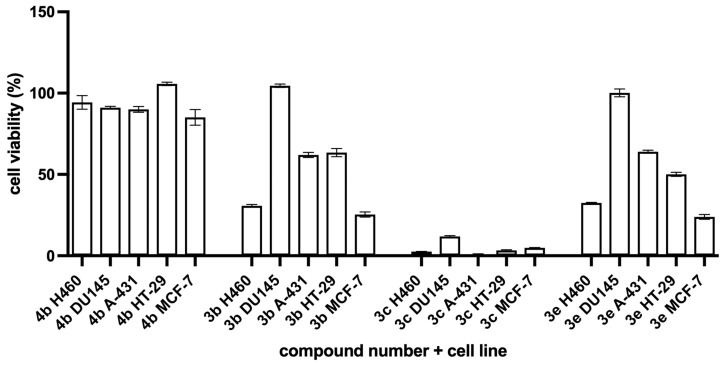
Bar graph representation of cancer cell line viability after treatment with tetrahydroquinolines.

The potent activity of the 1,2,3,4-tetrahydroquinolines in Figure 5 shows that incorporating an aryl group into position 4 of the quinoline structure dramatically increased the antiproliferative effect by up to 90% compared to the parent compound **4**. Compound **3c** showed the greatest antiproliferative effect with an unsubstituted phenyl ring at the 4 position. Adding a substituent to this ring led to a total loss of activity in the DU145 prostate carcinoma cell lines. However, compounds **3b** and **3e** maintained a fairly good level of activity in the H460 lung carcinoma cell line, (30.7% and 32.5%, respectively) and in the MCF7 breast adenocarcinoma cell lines (25.4% and 23.9%, respectively). It is important to note that all the tetrahydroquinoline analogues tested (**3b**, **3c**, and **3e**) were still more effective than their original quinoline structures **4**.

Due to its notable biological activity, the lead compound **3c** was further tested across various concentrations in these cell lines. The compound demonstrated effective activity in the H460 lung carcinoma, A-431 skin carcinoma, and HT-29 colon adenocarcinoma cells, with IC_50_ values of 4.9 ± 0.7, 2.0 ± 0.9, and 4.4 ± 1.3 μM, respectively. Therefore, compound 3c showed its most potent inhibition effects against skin carcinoma cells. Although the IC_50_ values were marginally higher in the DU145 prostate carcinoma and MCF7 breast adenocarcinoma cells, they still indicated substantial levels of activity, with values at 12.0 ± 1.6 and 14.6 ± 3.9 μM, respectively.

## 3. Materials and Methods

- Analytical data

Melting points (uncorrected) were measured using a Mel-Temp melting point apparatus. The Microanalysis Unit of the University of Otago, New Zealand, performed microanalyses. Infrared spectra were recorded as Nujol mulls on a Perkin-Elmer 298 (Beaconsfield, UK) or a Perkin-Elmer 580B spectrometer. Ultraviolet–visible spectra were recorded in methanol (unless otherwise stated) on a Hitachi UV-3200 spectrometer (Hitachinaka, Japan). ^1^H and ^13^C NMR spectra were obtained for the designated solvents on a Bruker AC300F (300 MHz) spectrometer (Bruker Pty Ltd., Preston, NSW, Australia). ^1^H NMR data were recorded as follows: chemical shift measured in parts per million (ppm) downfield from TMS (δ), multiplicity, observed coupling constant (*J*) in Hertz (Hz), proton count. Multiplicities are reported as singlet (s), broad singlet (bs), doublet (d), triplet (t), quartet (q), quintet (quin), and multiplet (m). ^13^C NMR chemical shifts are reported in ppm downfield from TMS and identifiable carbons are given. The EI and ES mass spectra were recorded on an AEI MS 12 mass spectrometer (Washington, D.C., USA) at 70 eV ionizing potential and 8000 V accelerating voltage with an ion source temperature of 210 °C. Kieselgel 60H (Merck, Rahway, NJ, USA, Art 7736) was employed for flash chromatography and thin layer chromatography (TLC) was performed on DC Aluminium Foil Kieselgel F_254_ (Merck, Art 5554). Solvents and reagents were purified by literature methods. Petroleum ether refers to the hydrocarbon fraction of boiling range 60–80 °C. Compounds were detected by short and long ultraviolet light and with iodine vapor.

- Cell culture

MCF-7, H-460, A-431, and DU-145 cells were maintained in Dulbecco’s modified Eagle’s medium (Gibco, Life Technologies, Carlsbad, CA, USA) supplemented with 10% fetal bovine serum, while HT-29 was maintained in RPMI media (Gibco, Life Technologies) supplemented with 10% fetal bovine serum. All cell lines were cultured at 37 °C, under 5% CO_2_ in a humidified atmosphere.

- In vitro growth inhibition assays

In vitro growth inhibition assays were performed in triplicate using the Alamar Blue assay. Briefly, cells in logarithmic phase growth were seeded onto 96-well plates at densities as follows:

MCF-7 = 6000 cells/well, 6 h Alamar Blue incubation;

HT-29 = 650 cells/well, 3 h Alamar Blue incubation;

H-460 = 600 cells/well, 6 h Alamar Blue incubation;

A-431 = 2100 cells/well, 6 h Alamar Blue incubation;

DU-145 = 2000 cells/well, 6 h Alamar Blue incubation.

A Multidrop 384 (Thermo Scientific, Waltham, MA, USA) was used and cells were allowed to adhere. After 24 h of incubation, test compounds, positive control (25 µM thonzonium bromide), and vehicle only (DMSO) controls were added to duplicate wells using a Hamilton Nimbus robotic platform. After 72 h of drug exposure, metabolic activity was detected by addition of Alamar Blue reagent and determined by measurement of fluorescence intensity (excitation 555 nm, emission 585 nm) using a SpectraMax M5 (Molecular Devices, San Jose, CA, USA) plate reader. Percentage of cell viability was determined at a fixed drug concentration of 25 μM. A value of 0% is indicative of total cell growth inhibition. Compounds showing appreciable percentage growth inhibition underwent further dose response analysis allowing for the calculation of an IC_50_ value. This value is the drug concentration at which cell growth is inhibited to its half maximal value.

### 3.1. General Procedures

Synthesis of 3,4-diaryl-5,7-dimethoxy-1,2,3,4-tetrahydroquinolin-4-ols (**5a**–**b**)

To a solution of azaisoflavone **4** (2.7 mmol) in anhydrous THF (10 mL) was slowly added phenylmagnesium bromide (5.5 mmol) in an atmosphere of nitrogen. The reaction mixture was heated at reflux for 5 h, then quenched with NH_4_Cl solution (25 mL, 20%) and extracted with ethyl acetate (2 × 50 mL). The combined organic layers were washed with brine (25 mL), dried over anhydrous Na_2_SO_4_, and concentrated under vacuum. The crude product was then purified using column chromatography (8% ethyl acetate in *n*-hexane) to give 3,4-diaryl-azaisoflavan-4-ol **5** as a white solid.

Synthesis of 3,4-diaryl-5,7-dimethoxy-quinolines (**6a**–**b**)

To a solution of 3,4-diaryl-5,7-dimethoxy-1,2,3,4-tetrahydroquinolin-4-ol **5** (2 mmol) in DCM (25 mL) was added 5 drops of BF_3_·OEt_2_ at room temperature. The reaction mixture was further stirred at room temperature for 4 h. After the completion of the reaction, it was quenched by the addition of a saturated solution of NaHCO_3_ (25 mL). The organic layer was collected, dried over anhydrous Na_2_SO_4_, and concentrated under vacuum. The crude solid was purified using column chromatography (10% ethyl acetate in *n*-hexane) to give 3,4-diaryl-5,7-dimethoxy quinoline **6** as a white solid.

Synthesis of 3,4-diaryl-5,7-dimethoxy-quinolines (**6c**–**e**)

The suspension of ethyl 3,4-diaryl-5,7-dimethoxy quinoline-1(2*H*)-carboxylate **9** (0.45 mmol) in HBr in AcOH (2 mL) was stirred at room temperature overnight. The reaction was quenched with ice water and the precipitate was filtered and washed with cold water. The crude solid was purified by column chromatography (10% ethyl acetate in *n*-hexane) to give the title compound **6** as a white solid (0.08 g, 46%).

Synthesis of Ethyl 3,4-diaryl-4-hydroxy-5,7-dimethoxy-3,4-dihydroquinoline-1(2*H*)-carboxylates (**8a**–**g**)

To a solution of azaisoflavone **7** (3.45 mmol) in anhydrous THF (15 mL) was slowly added phenylmagnesium bromide (6.9 mmol) in an atmosphere of nitrogen. The reaction mixture was heated at reflux for 4 h, then quenched with NH_4_Cl solution (25 mL, 20%) and extracted with ethyl acetate (2 × 50 mL). The combined organic layers were washed with brine (25 mL), dried over anhydrous Na_2_SO_4_, and concentrated under vacuum. The crude product was purified using column chromatography (4% ethyl acetate in *n*-hexane) to give the title compound **8** as a white solid.

Synthesis of Ethyl 3,4-diaryl-5,7-dimethoxy-quinoline-1(2*H*)-carboxylates (**9a**–**g**)

To a solution of 3,4-diaryl-4-hydroxy-5,7-dimethoxy-quinoline-1(2*H*)-carboxylate **8** (2.9 mmol) in DCM (25 mL) were added 5 drops of BF_3_·OEt_2_. The reaction mixture was stirred at room temperature for 4 h. After the completion of reaction, it was quenched by the addition of saturated solution of NaHCO_3_ (25 mL). The organic layer was collected, dried over anhydrous Na_2_SO_4_, and concentrated under vacuum. The crude solid was purified by column chromatography (3% ethyl acetate in *n*-hexane) to give the title compound **9** as a white solid.

Synthesis of -Ethyl *cis*-3,4-diaryl-5,7-dimethoxy-3,4-dihydroquinolin-1(2*H*)-carboxylates (**10b**–**g**)

To a solution of ethyl 3,4-diaryl-5,7-dimethoxy-quinoline-1(2*H*)-carboxylate **9** (1.35 mmol) in THF (10 mL) was added 10% palladium charcoal (0.2 g). The mixture was hydrogenated at atmospheric pressure for 24 h. The catalyst was removed by filtration through Celite^®^ and the filtrate was concentrated under vacuum. The crude solid was purified using column chromatography (2% ethyl acetate in *n*-hexane) to give the title compound **10** as a white solid.

Synthesis of *cis*-3,4-diaryl-5,7-dimethoxy-1,2,3,4-tetrahydroquinolines (**3b**–**g**)

The suspension of ethyl *cis*-3,4-diaryl-5,7-dimethoxy-3,4-dihydroquinoline-1(2*H*)-carboxylate **10** (1 mmol) was heated at reflux in 10% NaOH in ethanol (10 mL) for 8 h. After the completion of reaction, ethanol was concentrated under vacuum. The residue was taken in ethyl acetate (50 mL), washed with water (20 mL), dried over anhydrous Na_2_SO_4_, and concentrated under vacuum. The crude solid was purified using column chromatography (2% ethyl acetate in *n*-hexane) to give the title compound **3** as a white solid.

Synthesis of 5,7-dihydroxy-3-(4-hydroxyphenyl)quinoline (**12**)

The solution of 5,7-dimethoxy-3-(4-methoxyphenyl)-4-phenyl-1,2,3,4-tetrahydroquinoline **3c** (0.2 g, 0.5 mmol) in chlorobenzene (5 mL) was heated at reflux with aluminum chloride (0.2 g, 1.6 mmol) for 1 h. The reaction mixture was then quenched with ice-water to give a yellow precipitate. The crude solid was purified using column chromatography (15% ethyl acetate in *n*-hexane) to give the title compound **12** as a brown solid.

Synthesis of 3,4-diaryl-5-hydroxy-7-methoxy-1,2,3,4-tetrahydroquinolines (**13d,f,g**)

To a solution of 3,4-diaryl-5,7-dimethoxy-1,2,3,4-tetrahydroquinoline **3** (0.55 mmol) in DCM (5 mL) was added boron tribromide (1.65 mmol). The reaction mixture was further stirred at room temperature for 1 h, quenched with MeOH (2 mL), and concentrated under vacuum. The crude residue was taken in DCM (50 mL), washed with sat. NaHCO_3_ (10 mL), dried over anhydrous Na_2_SO_4_, and concentrated under vacuum. The crude solid was purified using column chromatography (8% ethyl acetate in *n*-hexane) to give title compound **13** as a yellow solid.

### 3.2. Experimental Data

3-(4-Bromophenyl)-5,7-dimethoxy-4-phenyl-1,2,3,4-tetrahydroquinolin-4-ol (**5a**)

White solid (0.9 g, 77%). M.p. 164–166 °C; UV (MeOH): λ_max_ 201 (ε 49,266 cm^−1^M^−1^), 221 (68532) nm; IR (KBr): ν_max_ 3367, 3007, 2944, 1605, 1589, 1501, 1487, 1201, 1149, 1132, 1075, 1061, 994, 819, 751, 721, 699 cm^−1^; ^1^H NMR (300 MHz, CDCl_3_): δ 3.12 (dd, *J* = 3.0, 9.0 Hz, 1H, H2a), 3.24 (dd, *J* = 3.0, 9.0 Hz, 1H, H2b), 3.35 (s, 3H, OMe), 3.43–3.49 (m, 1H, H3), 3.79 (s, 4H, OMe, OH), 4.14 (bs, 1H, NH), 5.87 (s, 2H, ArH6, ArH8), 6.90 (d, *J* = 9.0 Hz, 2H, ArH2′, ArH6′), 7.11–7.20 (m, 5H, Ph), 7.30 (d, *J* = 9.0 Hz, 2H, ArH3′, ArH5′); ^13^C NMR (75.6 MHz, CDCl_3_): δ 43.7 (CH_2_NH), 53.4 (CHAr′), 55.1 (OMe), 55.2 (OMe), 74.0 (C-OH), 90.2 (ArC6), 91.6 (ArC8), 108.5 (ArC), 120.3 (ArC4′), 125.5 (ArC2″, ArC6″), 126.0 (ArC4″), 127.2 (ArC3″, ArC5″), 130.3 (ArC2′, ArC6′), 131.4 (ArC3′, ArC5′), 146.9 (ArC1″), 150.0 (ArC1′, ArC), 159.8 (ArC7), 160.7 (ArC5); HRMS (ESI) *m*/*z* Calcd. for C_23_H_23_BrNO_3_ (M + H)^+^ 440.0861. Found 440.0848; Anal. Calcd. for C_23_H_22_BrNO_3_: C, 62.74; H, 5.04; N, 3.18. Found: C, 62.97; H, 5.14; N, 3.14.

5,7-Dimethoxy-3,4-bis(4-methoxyphenyl)-1,2,3,4-tetrahydroquinolin-4-ol (**5b**)

White solid (1.4 g, 72.3%). M.p. 172–174 °C; UV (MeOH): λ_max_ 203 (ε 58,256 cm^−1^M^−1^), 222 (58631) nm; IR (KBr): ν_max_ 3388, 3000, 2938, 2838, 1603, 1585, 1508, 1201, 1180, 1165, 1149, 1132, 1067, 1025, 824, 751, 728, 701 cm^−1^; ^1^H NMR (300 MHz, CDCl_3_): δ 2.85 (dd, *J* = 3.0, 12.0 Hz, 1H, H2a), 2.91 (dt, *J* = 3.0, 12.0 Hz, 1H, H2b), 3.16 (s, 3H, OMe), 3.40–3.46 (m, 1H, H3), 3.64 (s, 3H, OMe), 3.65 (s, 3H, OMe), 3.66 (s, 3H, OMe), 4.60 (s, 1H, OH), 5.69 (d, *J* = 3.0 Hz, 1H, ArH6), 5.85 (d, *J* = 3.0 Hz, 1H, ArH8), 6.08 (bs, 1H, NH), 6.62 (m, 4H, ArH2′, ArH3′, ArH5′, ArH6′), 6.78 (d, *J* = 9.0 Hz, 2H, ArH2″, ArH6″), 6.87 (d, *J* = 9.0 Hz, 2H, ArH2″, ArH6″); ^13^C NMR (75.6 MHz, CDCl_3_): δ 43.1 (CH_2_NH), 53.6 (CHAr′), 55.0 (OMe), 55.1 (2 × OMe), 55.3 (OMe), 73.1 (C-OH), 89.0 (ArC6), 91.5 (ArC8), 108.9 (ArC), 112.3 (ArC3′, ArC5′), 112.6 (ArC3″, ArC5″), 126.6 (ArC2′, ArC6′), 130.8 (ArC2″, ArC6″), 132.4 (ArC1″), 143.0 (ArC1′), 147.9 (ArC), 157.0 (ArC4′), 157.7 (ArC4″), 160.2 (ArC7), 160.2 (ArC5); HRMS (ESI) *m*/*z* Calcd. for C_25_H_28_NO_5_ (M + H)^+^: 422.1967. Found: 422.1953.

3-(4-Bromophenyl)-5,7-dimethoxy-4-phenylquinoline (**6a**)

White solid (0.55 g, 66%). M.p. 192–194 °C; UV (MeOH): λ_max_ 207 (ε 61,432 cm^−1^M^−1^), 255 (53,581), 343 (3880) nm; IR (KBr): ν_max_ 2961, 1609, 1578, 1366, 1204, 1150, 1082, 1040, 829, 771, 753, 723, 708, 692 cm^−1^; ^1^H NMR (300 MHz, CDCl_3_): δ 3.38 (s, 3H, OMe), 3.96 (s, 3H, OMe), 6.46 (d, *J* = 3.0 Hz, 1H, ArH6), 6.92 (d, *J* = 9.0 Hz, 2H, ArH2′, ArH6′), 7.00–7.04 (m, 2H, Ph), 7.13 (d, *J* = 3.0 Hz, 1H, ArH8), 7.17–7.19 (m, 3H, Ph), 7.25 (d, *J* = 9.0 Hz, 2H, ArH3′, ArH5′), 8.75 (s, 1H, H2); ^13^C NMR (75.6 MHz, CDCl_3_): δ 55.3 (OMe), 55.5 (OMe), 99.8 (ArC6), 100.4 (ArC8), 114.5 (ArC), 120.8 (ArC3), 126.3 (ArC3′, ArC5′), 126.6 (ArC4′, ArC1′), 129.0 (ArC4″), 130.7 (ArC2′, ArC6′), 131.8 (ArC2″, ArC6″), 137.4 (ArC3″, ArC5″), 139.9 (ArC4), 144.9 (ArC1″), 150.7 (ArC), 151.2 (ArC2), 157.6 (ArC7), 160.9 (ArC5); HRMS (ESI) *m*/*z* Calcd. for C_23_H_19_BrNO_2_ (M + H)^+^ 420.0599. Found: 420.0584; Anal. Calcd. for C_23_H_18_BrNO_2_: C, 65.73; H, 4.32; N, 3.33. Found: C, 65.79; H, 4.34; N, 3.27.

5,7-Dimethoxy-3,4-bis(4-methoxyphenyl)quinoline (**6b**)

White solid (0.7 g, 54%). M.p. 180–182 °C; UV (MeOH): λ_max_ 202 (ε 380,769 cm^−1^M^−1^), 206 (380,769), 212 (384,615), 256 (280,769), 341 (65,384) nm; IR (KBr): ν_max_ 2935, 2837, 1610, 1578, 1555, 1510, 1450, 1408, 1365, 1330, 1286, 1203, 1147, 1117, 1042, 1027, 826, 801, 787, 722, 656 cm^−1^; ^1^H NMR (300 MHz, CDCl_3_): δ 3.43 (s, 3H, OMe), 3.76 (s, 3H, OMe), 3.79 (s, 3H, OMe), 3.96 (s, 3H, OMe), 6.46 (d, *J* = 3.0 Hz, 1H, ArH6), 6.71–6.75 (m, 4H, ArH3′, ArH5′, ArH3″, ArH5″), 6.93–6.98 (m, 4H, ArH2′, ArH6′, ArH2″, ArH6″), 7.12 (d, *J* = 3.0 Hz, 1H, ArH8), 8.75 (s, 1H, H2); ^13^C NMR (75.6 MHz, CDCl_3_): δ 55.1 (OMe), 55.2 (OMe), 55.5 (OMe), 55.6 (OMe), 99.8 (ArC6, ArC8), 100.5 (ArC), 112.1 (ArC3′, ArC5′), 112.2 (ArC3″, ArC5″), 113.2 (ArC1′), 115.0 (ArC), 130.3 (ArC1″), 130.4 (ArC2′), 131.0 (ArC6′), 131.4 (ArC2″), 132.5 (ArC6″), 132.8 (ArC4), 150.5 (ArC), 152.0 (ArC2), 157.8 (ArC7), 157.9 (ArC5), 158.1 (ArC4′), 160.5 (ArC4″); HRMS (ESI) *m*/*z* Calcd. for C_25_H_24_NO_4_ (M + H)^+^: 402.1705. Found: 402.1692.

5,7-Dimethoxy-3-(4-methoxyphenyl)-4-phenylquinoline (**6c**)

White solid (0.08 g, 46%). M.p. 216–218 °C; UV (MeOH): λ_max_ 209 (ε 70,446 cm^−1^M^−1^), 257 (73,791), 347 (4089) nm; IR (KBr): ν_max_ 2961, 2837, 1614, 1510, 1366, 1240, 1203, 1149, 1116, 1041, 1026, 837, 829, 767, 700 cm^−1^; ^1^H NMR (300 MHz, CDCl_3_): δ 3.38 (s, 3H, OMe), 3.74 (s, 3H, OMe), 3.96 (s, 3H, OMe), 6.45 (d, *J* = 3.0 Hz, 1H, ArH6), 6.70 (d, *J* = 9.0 Hz, 2H, ArH2′, ArH6′), 6.97 (d, *J* = 9.0 Hz, 2H, ArH3′, ArH5′), 7.02–7.06 (m, 2H, Ph), 7.13 (d, *J* = 3.0 Hz, 1H, ArH8), 7.17–7.19 (m, 3H, Ph), 8.76 (s, 1H, ArH2); ^13^C NMR (75.6 MHz, CDCl_3_): δ 55.0 (OMe), 55.3 (OMe), 55.4 (OMe), 99.7 (ArC6, ArC8), 100.4 (ArC), 113.0 (ArC5′, ArC3′), 114.6 (ArC1′), 126.0 (ArC3), 126.5 (ArC2″, ArC6″), 129.0 (ArC3″, ArC5″), 130.7 (ArC4″), 131.3 (ArC2′, ArC6′), 132.0 (ArC4), 140.4 (ArC1″), 144.6 (ArC), 150.3 (ArC2), 157.6 (ArC4′), 158.1 (ArC7), 160.5 (ArC5); HRMS (ESI) *m*/*z* Calcd. for C_24_H_22_NO_3_Na (M + Na)^+^: 393.1979. Found: 393.1968; Anal. Calcd. for C_24_H_21_NO_3_: C, 77.61; H, 5.70; N, 3.77. Found: C, 77.38; H, 5.81; N, 3.73.

4-(4-(*tert*-Butyl)phenyl)-5,7-dimethoxy-3-(4-methoxyphenyl)quinoline (**6d**)

White solid (0.06 g, 38%). M.p. 172–174 °C; UV (MeOH): λ_max_ 205 (ε 48,702 cm^−1^M^−1^), 257 (34,961) nm; IR (KBr): ν_max_ 2923, 2853, 1615, 1511, 1449, 1204, 1148, 1125, 1105, 1030, 824, 591 cm^−1^; ^1^H NMR (300 MHz, CDCl_3_): δ 1.30 (s, 9H, 3 × Me), 3.36 (s, 3H, OMe), 3.74 (s, 3H, OMe), 3.96 (s, 3H, OMe), 6.45 (d, *J* = 3.0 Hz, 1H, ArH6), 6.70 (d, *J* = 9.0 Hz, 2H, ArH3′, ArH5′), 6.93–6.99 (m, 4H, ArH2″, ArH3″, ArH5″, ArH6″), 7.14 (d, *J* = 3.0 Hz, 1H, ArH8), 7.20 (d, *J* = 8.4 Hz, 2H, ArH2′, ArH6′), 8.77 (s, 1H, ArH2); ^13^C NMR (75.6 MHz, CDCl_3_): δ 30.1 (MeC), 31.5 (MeC), 32.0 (MeC), 34.5 (CMe), 55.2 (2 × OMe), 55.6 (OMe), 100.0 (ArC6, ArC8), 100.4 (ArC), 113.2 (ArC3′, ArC5′), 117.8 (ArC1′), 122.9 (CHAr′), 123.5 (ArC2″, ArC6″), 128.9 (ArC3″, ArC5″), 131.0 (ArC2′, ArC6′), 131.5 (ArC1′), 132.3 (ArC4), 137.5 (ArC), 149.1 (ArC2), 151.9 (ArC4″), 157.9 (ArC5), 158.2 (ArC7), 160.7 (ArC4′); HRMS (ESI) *m*/*z* Calcd. for C_28_H_30_NO_3_ (M + H)^+^: 428.2226. Found: 428.2213.

5,7-Dimethoxy-3-(4-methoxyphenyl)-4-(*p*-tolyl)quinoline (**6e**)

White solid (0.08 g, 46.5%). M.p. 186–188 °C; UV (MeOH): λ_max_ 211 (ε 70,077 cm^−1^M^−1^), 256 (48,353), 340 (11,212) nm; IR (KBr): ν_max_ 2954, 1614, 1511, 1449, 1239, 1203, 1150, 1040, 1030, 840, 832, 811, 719, 582 cm^−1^; ^1^H NMR (300 MHz, CDCl_3_): δ 2.24 (s, 3H, MeAr″), 3.32 (s, 3H, OMe), 3.67 (s, 3H, OMe), 3.88 (s, 3H, OMe), 6.37 (d, *J* = 2.4 Hz, 1H, ArH6), 6.63 (d, *J* = 8.7 Hz, 2H, ArH3′, ArH5′), 6.84 (d, *J* = 8.1 Hz, 2H, ArH3″, ArH5″), 6.88–6.92 (m, 4H, ArH2′, ArH6′, ArH2″, ArH6″), 7.05 (d, *J* = 2.1 Hz, 1H, ArH8), 8.67 (s, 1H, ArH2); ^13^C NMR (75.6 MHz, CDCl_3_): δ 21.2 (MeAr″), 55.1 (OMe), 55.2 (OMe), 55.5 (OMe), 99.8 (ArC6, ArC8), 100.5 (ArC), 113.1 (ArC3′, ArC5′), 113.2 (ArC1′, ArC3), 127.2 (ArC2″, ArC6″), 127.3 (ArC3″, ArC5″), 129.0 (ArC2′, ArC6′), 131.4 (ArC1″, ArC4″), 135.4 (ArC4), 144.9 (ArC), 150.5 (ArC2), 157.8 (ArC5), 158.1 (ArC7), 160.5 (ArC4′); HRMS (ESI) *m*/*z* Calcd. for C_25_H_24_NO_3_ (M + H)^+^: 386.1756. Found: 386.1744.

Ethyl 3-(4-bromophenyl)-4-hydroxy-5,7-dimethoxy-4-phenyl-3,4-dihydroquinoline-1(2*H*)-carboxylate (**8a**)

White solid (1.6 g, 88.5%). M.p. 144–146 °C; UV (MeOH): λ_max_ 219 (ε 64,000 cm^−1^M^−1^), 247 (16,666), 336 (7333) nm; IR (KBr): ν_max_ 3532, 2982, 1706, 1576, 1488, 1455, 1384, 1304, 1237, 1213, 1066, 1042, 1010, 823, 759, 723, 704 cm^−1^; ^1^H NMR (300 MHz, CDCl_3_): δ 1.19 (t, *J* = 6.0 Hz, 3H, CH_3_CH_2_), 3.02 (dd, *J* = 3.0, 12.0 Hz, 1H, H2a), 3.19 (s, 3H, OMe), 3.82 (t, *J* = 12.0 Hz, 1H, H3), 3.74 (s, 3H, OMe), 4.11–4.18 (m, 3H, CH_2_CH_3_, H2b), 6.28 (d, *J* = 3.0 Hz, 1H, ArH6), 6.63 (d, *J* = 9.0 Hz, 2H, ArH2′, ArH6′), 6.89–6.92 (m, 2H, Ph), 7.00 (d, 3.0 Hz, 1H, ArH8), 7.07–7.14 (m, 3H, Ph), 7.27 (d, *J* = 9.0 Hz, 2H, ArH3′, ArH5′); ^13^C NMR (75.6 MHz, CDCl_3_): δ 14.4 (Me), 46.0 (CH_2_N), 54.5 (CHAr′), 55.3 (OMe), 55.5 (OMe), 62.1 (OCH_2_), 74.5 (C-OH), 97.0 (ArC6, ArC8), 100.1 (ArC), 116.7 (ArC4′), 120.7 (ArC4″), 125.1 (ArC2″, ArC6″), 126.2 (ArC5″), 127.2 (ArC3″), 130.3 (ArC2′, ArC6′), 131.5 (ArC3′, ArC5′), 136.2 (ArC), 140.0 (ArC1′), 148.7 (ArC1″), 154.1 (COO), 158.5 (ArC7), 159.5 (ArC5); HRMS (ESI) *m*/*z* Calcd. for C_26_H_26_BrNO_5_Na (M + Na)^+^: 534.0892. Found: 534.0881; Anal. Calcd. for C_26_H_26_BrNO_5_: C, 60.95; H, 5.11; N, 2.73. Found: C, 60.94; H, 5.18; N, 2.69.

Ethyl 4-hydroxy-5,7-dimethoxy-3,4-bis(4-methoxyphenyl)-3,4-dihydroquinoline-1(2*H*)-carboxylate (**8b**)

White solid (1.6 g, 84.5%). M.p. 108–110 °C; UV (MeOH): λ_max_ 220 (ε 56,085 cm^−1^M^−1^), 276 (3365), 337 (5608) nm; IR (KBr): ν_max_ 3464, 2934, 1677, 1610, 1582, 1509, 1172, 1144, 1062, 1027, 956, 891, 826, 765 cm^−1^; ^1^H NMR (300 MHz, CDCl_3_): δ 1.24 (t, *J* = 6.0 Hz, 3H, CH_3_CH_2_), 3.06 (dd, *J* = 3.0, 12.0 Hz, 1H, H2a), 3.36 (s, 3H, OMe), 3.77 (s, 6H, 2 × OMe), 3.83 (dd, *J* = 3.0, 9.0 Hz, 1H, H2b), 3.84 (s, 3H, OMe), 4.19 (q, *J* = 6.0 Hz, 2H, CH_2_CH_3_), 4.28 (dd, *J* = 3.0, 12.0 Hz, 1H, H3), 6.23 (d, *J* = 3.0 Hz, 1H, ArH6), 6.69 (d, *J* = 9.0 Hz, 2H, ArH3′, ArH5′), 6.73–6.82 (m, 4H, ArH2″, ArH3″, ArH5″, ArH6″), 6.90 (d, *J* = 9.0 Hz, 2H, ArH2′, ArH6′), 7.16 (d, *J* = 3.0 Hz, 1H, ArH8); ^13^C NMR (75.6 MHz, CDCl_3_): δ 14.4 (Me), 46.5 (CH_2_N), 54.3 (OMe), 55.0 (OMe), 55.3 (2 × OMe), 55.6 (CHAr′), 61.9 (OCH_2_), 74.5 (C-OH), 97.0 (ArC6, ArC8), 100.0 (ArC), 112.4 (ArC3′, ArC5′), 112.7 (ArC3″, ArC5″), 117.0 (ArC), 126.3 (ArC2′, ArC6′), 130.7 (ArC2″, ArC6″), 140.1 (ArC1″), 141.4 (ArC1′), 154.2 (COO), 157.7 (ArC4″), 158.2 (ArC4′), 158.6 (ArC7), 159.3 (ArC5); HRMS (ESI) *m*/*z* Calcd. for C_28_H_31_NO_7_Na (M + Na)^+^: 516.1998. Found: 516.1981.

Ethyl 4-hydroxy-5,7-dimethoxy-3-(4-methoxyphenyl)-4-phenyl-3,4-dihydroquinoline-1(2*H*)-carboxylate (**8c**)

White solid (1.5 g, 85%). M.p. 140–142 °C; UV (MeOH): λ_max_ 219 (ε 41,395 cm^−1^M^−1^), 337 (3255) nm; IR (KBr): ν_max_ 3561, 2971, 1699, 1607, 1577, 1385, 1302, 1237, 1178, 1042, 1008, 829, 765, 752, 736, 703 cm^−1^; ^1^H NMR (300 MHz, CDCl_3_): δ 1.26 (t, *J* = 6.0 Hz, 3H, CH_3_CH_2_), 3.08 (dd, *J* = 3.0, 9.0 Hz, 1H, H2a), 3.32 (s, 3H, OMe), 3.77 (s, 3H, OMe), 3.83 (dd, *J* = 3.0 Hz, 9.0 Hz, 1H, H2b), 3.84 (s, 3H, OMe), 4.21 (q, *J* = 6.0 Hz, 2H, CH_2_CH_3_), 4.33 (dd, *J* = 3.0 Hz, 12.0 Hz, 1H, H3), 6.23 (d, *J* = 3.0 Hz, 1H, ArH6), 6.72–6.79 (m, 4H, ArH3′, ArH5′, ArH3″, ArH5″), 6.97–7.00 (m, 2H, ArH4″, ArH8), 7.13–7.18 (m, 4H, ArH2′, ArH6′, ArH2″, ArH6″); ^13^C NMR (75.6 MHz, CDCl_3_): δ 14.4 (Me), 54.2 (CH_2_N), 54.9 (CHAr′), 55.0 (OMe), 55.3 (OMe), 55.5 (OMe), 61.9 (OCH_2_), 74.7 (C-OH), 96.5 (ArC6), 97.0 (ArC8), 100.1 (ArC), 112.6 (ArC3′, ArC5′), 125.2 (ArC3″), 125.5 (ArC5″), 125.9 (ArC4″), 127.1 (ArC2′, ArC6′), 129.2 (ArC2″), 129.6 (ArC6″), 140.1 (ArC), 140.6 (ArC1′), 149.1 (ArC1″), 154.2 (COO), 157.4 (ArC4′), 157.7 (ArC7), 158.3 (ArC5); HRMS (ESI) *m*/*z* Calcd. for C_27_H_29_NO_6_Na (M + Na)^+^: 486.1893. Found: 486.1881; Anal. Calcd. for C_27_H_29_NO_6_: C, 69.96; H, 6.31; N, 3.02. Found: C, 69.75; H, 6.37; N, 3.01.

Ethyl 4-(4-(*tert*-butyl)phenyl)-4-hydroxy-5,7-dimethoxy-3-(4-methoxyphenyl)-3,4-dihydroquinoline-1(2*H*)-carboxylate (**8d**)

White solid (1.6 g, 80%). M.p. 118–120 °C; UV (MeOH): λ_max_ 202 (ε 102,479 cm^−1^M^−1^), 222 (113,813) nm; IR (KBr): ν_max_ 3551, 2957, 1703, 1584, 1515, 1237, 1140, 1060, 1037, 827, 756, 736 cm^−1^; ^1^H NMR (300 MHz, CDCl_3_): δ 1.23 (t, *J* = 6.0 Hz, 3H, CH_3_CH_2_), 1.27 (s, 9H, 3 × Me), 3.13 (dd, *J* = 2.1, 10.2 Hz, 1H, H2a), 3.35 (s, 3H, OMe), 3.79 (s, 3H, OMe), 3.86 (s, 3H, OMe), 4.10–4.19 (m, 3H, CH_2_CH_3_, H2b), 4.24 (dd, *J* = 2.7, 12.9 Hz,1H, H3), 6.26 (d, *J* = 3.0 Hz, 1H, ArH6), 6.76 (d, *J* = 9.0 Hz, 2H, ArH2′, ArH6′), 6.84 (d, *J* = 9.0 Hz, 2H, ArH3′, ArH5′), 6.94 (d, *J* = 8.4 Hz, 2H, ArH2″, ArH6″), 7.17–7.20 (m, 3H, ArH8, ArH3″, ArH5″); ^13^C NMR (75.6 MHz, CDCl_3_): δ 14.5 (Me), 31.3 (MeC), 31.4 (MeC), 34.3 (MeC), 46.7 (CMe), 54.0 (CH_2_N), 55.1 (OMe), 55.4 (OMe), 55.7 (OMe), 60.4 (CHAr′), 62.9 (OCH_2_), 74.8 (C-OH), 97.2 (ArC6, ArC8), 100.3 (ArC), 112.8 (ArC3′, ArC5′), 124.0 (ArC3″, ArC5″), 125.0 (ArC2′, ArC6′), 129.7 (ArC), 130.7 (ArC2″, ArC6″), 140.2 (ArC1′), 146.2 (ArC1″), 148.9 (ArC4″), 154.4 (COO), 158.3 (ArC4′), 158.8 (ArC7), 159.4 (ArC5); HRMS (ESI) *m*/*z* Calcd. for C_31_H_37_NO_6_Na (M + Na)^+^: 542.2519. Found: 542.2496.

Ethyl 4-hydroxy-5,7-dimethoxy-3-(4-methoxyphenyl)-4-(*p*-tolyl)-3,4-dihydroquinoline-1(2*H*)-carboxylate (**8e**)

White solid (1.5 g, 82%). M.p. 108–110 °C; UV (MeOH): λ_max_ 220 (ε 47,281 cm^−1^M^−1^), 336 (5673) nm; IR (KBr): ν_max_ 3470, 2935, 2838, 1684, 1611, 1584, 1511, 1374, 1313, 1179, 1143, 1057, 1029, 956, 829, 817, 764 cm^−1^; ^1^H NMR (300 MHz, CDCl_3_): δ 1.16 (t, *J* = 6.0 Hz, 3H, CH_3_CH_2_), 2.22 (s, 3H, MeAr″), 2.99 (dd, *J* = 1.8, 10.8 Hz, 1H, H2a), 3.27 (s, 3H, OMe), 3.69 (s, 3H, OMe), 3.71 (s, 1H, OH), 3.76 (s, 3H, OMe), 3.77 (dd, *J* = 2.1, 10.5 Hz, 1H, H2b), 4.11 (q, *J* = 6.0 Hz, 2H, CH_2_CH_3_), 4.19 (dd, *J* = 2.7, 12.6 Hz, 1H, H3), 6.15 (d, *J* = 3.0 Hz, 1H, ArH6), 6.65–6.75 (m, 4H, ArH3′, ArH5′, ArH3″, ArH5″), 6.79 (d, *J* = 8.1 Hz, 2H, ArH2′, ArH6′), 6.88 (d, *J* = 8.1 Hz, 2H, ArH2″, ArH6″), 7.08 (d, *J* = 3.0 Hz, 1H, ArH8); ^13^C NMR (75.6 MHz, CDCl_3_): δ 14.5 (Me), 21.0 (MeAr″), 46.6 (CH_2_N), 55.3 (OMe), 55.4 (OMe), 55.6 (OMe), 55.7 (CHAr′), 62.0 (OCH_2_), 74.8 (C-OH), 97.1 (ArC6), 97.2 (ArC8), 100.2 (ArC), 112.8 (ArC3′, ArC5′), 125.3 (ArC2′, ArC6′), 128.0 (ArC2″, ArC6″), 129.4 (ArC3″, ArC5″), 130.7 (ArC4″), 135.4 (ArC), 140.2 (ArC1′), 144.8 (ArC1″), 154.4 (COO), 158.7 (ArC4′), 159.1 (ArC7), 159.4 (ArC5); HRMS (ESI) *m*/*z* Calcd. for C_28_H_31_NO_6_Na (M + Na)^+^: 500.2049. Found: 500.2035.

Ethyl 4-hydroxy-5,7-dimethoxy-3,4-diphenyl-3,4-dihydroquinoline-1(2*H*)-carboxylate (**8f**)

White solid (1.55 g, 85%). M.p. 142–144 °C; UV (MeOH): λ_max_ 219 (ε 42,936 cm^−1^M^−1^), 243 (12,881), 337 (4293) nm; IR (KBr): ν_max_ 3232, 1614, 1511, 1449, 1239, 1203, 1150, 1040, 1030, 840, 832, 811, 719, 582 cm^−1^; ^1^H NMR (300 MHz, CDCl_3_): δ 1.29 (t, *J* = 6.0 Hz, 3H, CH_3_CH_2_), 3.17 (dd, *J* = 2.1, 10.8 Hz, 1H, H2a), 3.35 (s, 3H, OMe), 3.85 (d, *J* = 1.3 Hz, 1H, H2b), 3.88 (s, 3H, OMe), 3.93 (s, 1H, OH), 4.25 (q, *J* = 7.2 Hz, 2H, CH_2_CH_3_), 4.40 (dd, *J* = 2.7, 12.6 Hz, 1H, H3), 6.27 (d, *J* = 3.0 Hz, 1H, ArH6), 6.88–6.91 (m, 2H, ArH′), 7.02–7.05 (m, 2H, ArH″), 7.18–7.24 (m, 7H, ArH′, ArH″, ArH8); ^13^C NMR (75.6 MHz, CDCl_3_): δ 14.5 (Me), 46.4 (CH_2_N), 55.1 (OMe), 55.4 (OMe), 55.6 (CHAr′), 62.1 (OCH_2_), 74.9 (C-OH), 97.1 (ArC6, ArC8), 100.2 (ArC), 117.2 (ArC4′), 125.3 (ArC4″), 126.1 (ArC6′), 126.7 (ArC2′), 127.1 (ArC6″), 127.2 (ArC2″), 127.3 (ArC5′), 127.7 (ArC3′), 129.3 (ArC5″), 129.9 (ArC3″), 137.3 (ArC), 140.3 (ArC1′), 149.2 (ArC1″), 154.3 (COO), 158.6 (ArC7), 159.5 (ArC5); HRMS (ESI) *m*/*z* Calcd. for C_26_H_28_NO_5_ (M + H)^+^: 434.1967. Found: 434.1949; Anal. Calcd. for C_26_H_27_NO_5_: C, 72.04; H, 6.28; N, 3.23. Found: C, 72.20; H, 6.34; N, 3.19.

Ethyl 4-hydroxy-5,7-dimethoxy-3-phenyl-4-(*p*-tolyl)-3,4-dihydroquinoline-1(2*H*)-carboxylate (**8g**)

White solid (1.5 g, 78%). M.p. 170–172 °C; UV (MeOH): λ_max_ 219 (ε 66,666 cm^−1^M^−1^) nm; IR (KBr): ν_max_ 3585, 2956, 2939, 2905, 1697, 1581, 1453, 1397, 1320, 1280, 1225, 1146, 1080, 1057, 1030, 953, 845, 813, 762, 700 cm^−1^; ^1^H NMR (300 MHz, CDCl_3_): δ 1.27 (t, *J* = 6.0 Hz, 3H, CH_3_CH_2_), 2.34 (s, 3H, MeAr″), 3.17 (dd, *J* = 2.7, 10.2 Hz, 1H, H2a), 3.29 (s, 1H, OH), 3.39 (s, 3H, OMe), 3.88 (s, 3H, OMe), 3.90 (dd, *J* = 2.1, 10.5 Hz, 1H, H2b), 4.22 (q, *J* = 7.2 Hz, 2H, CH_2_CH_3_), 4.35 (dd, *J* = 2.7, 12.9 Hz, 1H, H3), 6.28 (d, *J* = 3.0 Hz, 1H, ArH6), 6.89–7.02 (m, 6H, ArH′, ArH″), 7.21–7.24 (m, 4H, ArH′, ArH″, ArH8); ^13^C NMR (75.6 MHz, CDCl_3_): δ 14.5 (Me), 21.2 (MeAr″), 46.5 (CH_2_N), 55.1 (OMe), 55.4 (OMe), 55.7 (CHAr′), 62.1 (OCH_2_), 74.8 (C-OH), 97.1 (ArC6, ArC8), 100.2 (ArC), 126.6 (ArC4′), 127.3 (ArC2′, ArC6′), 127.7 (ArC2″, ArC6″), 127.8 (ArC5′), 128.5 (ArC3′), 129.1 (ArC5″), 129.9 (ArC3″), 135.5 (ArC4″), 137.5 (ArC), 140.2 (ArC1″), 146.2 (ArC1′), 154.4 (COO), 158.7 (ArC7), 159.4 (ArC5); HRMS (ESI) *m*/*z* Calcd. for C_27_H_29_NO_5_Na (M + Na)^+^: 470.1943. Found: 470.1927; Anal. Calcd. for C_27_H_29_NO_5_: C, 72.46; H, 6.53; N, 3.13. Found: C, 72.66; H, 6.61; N, 3.09.

Ethyl 3-(4-bromophenyl)-5,7-dimethoxy-4-phenylquinoline-1(2*H*)-carboxylate (**9a**)

White solid (0.8 g, 55%). M.p. 188–190 °C; UV (MeOH): λ_max_ 215 (ε 55,234 cm^−1^M^−1^), 247 (19,910), 329 (14,129) nm; IR (KBr): ν_max_ 2977, 1699, 1597, 1568, 1402, 1325, 1204, 1152, 1045, 1008, 831, 818, 766, 755, 701 cm^−1^; ^1^H NMR (300 MHz, CDCl_3_): δ 1.29 (t, *J* = 6.0 Hz, 3H, CH_3_CH_2_), 3.23 (s, 3H, OMe), 3.84 (s, 3H, OMe), 4.24 (q, *J* = 6.0 Hz, 2H, CH_2_CH_3_), 4.54 (s, 2H, CH_2_N), 6.19 (d, *J* = 3.0 Hz, 1H, ArH6), 6.80 (d, *J* = 6.0 Hz, 2H, ArH2′, ArH6′), 6.95 (d, *J* = 3.0 Hz, 1H, ArH8), 6.97–6.99 (m, 2H, Ph), 7.12–7.14 (m, 3H, Ph), 7.24 (d, *J* = 6.0 Hz, 2H, ArH3′, ArH5′); ^13^C NMR (75.6 MHz, CDCl_3_): δ 14.4 (Me), 48.5 (CH_2_N), 55.3 (OMe), 55.4 (OMe), 62.1 (OCH_2_), 96.5 (ArC6, ArC8), 100.3 (ArC), 113.8 (Ar′C4), 120.1 (ArC3), 125.9 (ArC2′, ArC6′), 127.2 (ArC3″, ArC5″), 129.0 (ArC2″, ArC6″), 130.1 (ArC4″), 130.8 (ArC3′, ArC5′), 133.3 (ArC4), 138.6 (ArC1′), 139.8 (ArC), 140.0 (ArC1″), 153.4 (COO), 157.6 (ArC7), 159.9 (ArC5); HRMS (ESI) *m*/*z* Calcd. for C_26_H_24_BrNO_4_Na (M + Na)^+^: 516.0786. Found: 516.0766; Anal. Calcd. for C_26_H_24_BrNO_4_: C, 63.17; H, 4.89; N, 2.83. Found: C, 63.37; H, 4.98; N, 2.80.

Ethyl 5,7-dimethoxy-3,4-bis(4-methoxyphenyl)quinoline-1(2*H*)-carboxylate (**9b**)

White solid (0.75 g, 52%). M.p. 148–150 °C; UV (MeOH): λ_max_ 251 (ε 16,507 cm^−1^M^−1^), 325 (11,279) nm; IR (KBr): ν_max_ 2966, 2935, 2841, 1704, 1607, 1569, 1508, 1446, 1324, 1244, 1219, 1150, 1050, 831, 819, 758 cm^−1^; ^1^H NMR (300 MHz, CDCl_3_): δ 1.28 (t, *J* = 6.0 Hz, 3H, CH_3_CH_2_), 3.28 (s, 3H, OMe), 3.74 (s, 3H, OMe), 3.76 (s, 3H, OMe), 3.83 (s, 3H, OMe), 4.23 (q, *J* = 6.0 Hz, 2H, CH_2_CH_3_), 4.53 (s, 2H, CH_2_N), 6.20 (d, *J* = 3.0 Hz, 1H, ArH6), 6.68 (d, *J* = 9.0 Hz, 4H, ArH3′, ArH5′, ArH3″, ArH5″), 6.88 (d, *J* = 9.0 Hz, 4H, ArH2′, ArH6′, ArH2″, ArH6″), 6.98 (d, *J* = 3.0 Hz, 1H, ArH8); ^13^C NMR (75.6 MHz, CDCl_3_): δ 14.4 (Me), 48.9 (CH_2_N), 55.0 (2 × OMe), 55.3 (OMe), 55.4 (OMe), 61.9 (OCH_2_), 96.6 (ArC6, ArC8), 100.2 (ArC), 112.5 (ArC3′, ArC5′), 113.0 (ArC3″, ArC5″), 114.3 (CHAr′), 129.6 (ArC2″, ArC6″), 130.2 (ArC2″, ArC6″), 131.3 (ArC4), 132.3 (ArC1′), 132.9 (ArC1″), 139.7 (ArC), 153.5 (COO), 157.5 (ArC4′, ArC4″), 157.6 (ArC7), 159.4 (ArC5); HRMS (ESI) *m*/*z* Calcd. for C_28_H_30_NO_6_ (M + H)^+^: 476.2073. Found: 476.2059.

Ethyl 5,7-dimethoxy-3-(4-methoxyphenyl)-4-phenylquinoline-1(2*H*)-carboxylate (**9c**)

White solid (0.8 g, 54%). M.p. 148–150 °C; UV (MeOH): λ_max_ 215 (ε 57,573 cm^−1^M^−1^), 247 (20,562), 327 (14,393) nm; IR (KBr): ν_max_ 2964, 2901, 2840, 1707, 1601, 1573, 1365, 1307, 1216, 1172, 1144, 1041, 1028, 825, 748, 703 cm^−1^; ^1^H NMR (300 MHz, CDCl_3_): δ 1.28 (t, *J* = 6.0 Hz, 3H, CH_3_CH_2_), 3.22 (s, 3H, OMe), 3.73 (s, 3H, OMe), 3.84 (s, 3H, OMe), 4.24 (q, *J* = 6.0 Hz, 2H, CH_2_CH_3_), 4.55 (s, 2H, CH_2_N), 6.19 (d, *J* = 3.0 Hz, 1H, ArH6), 6.65 (d, *J* = 9.0 Hz, 2H, ArH2′, ArH6′), 6.85 (d, *J* = 9.0 Hz, 2H, ArH3′, ArH5′), 6.99–7.01 (m, 3H, Ph, ArH8), 7.10–7.13 (m, 3H, Ph); ^13^C NMR (75.6 MHz, CDCl_3_): δ 14.4 (Me), 55.0 (2 × OMe), 55.3 (OMe), 61.9 (CH_2_N), 65.8 (OCH_2_), 96.5 (ArC6, ArC8), 100.2 (ArC), 113.0 (ArC5′), 114.2 (ArC3′), 125.5 (ArC3), 127.1 (ArC4″, ArC4), 129.2 (ArC3″, ArC5″), 129.6 (ArC2″, ArC6″), 131.7 (ArC2′, ArC6′), 132.1 (ArC1′), 139.6 (ArC), 140.6 (ArC1″), 153.5 (COO), 157.4 (ArC4′), 157.7 (ArC7), 159.5 (ArC5); HRMS (ESI) *m*/*z* Calcd. for C_27_H_27_NO_5_Na (M + Na)^+^: 468.1787. Found: 468.1773; Anal. Calcd. for C_27_H_27_NO_5_: C, 72.79; H, 6.11; N, 3.14. Found: C, 72.84; H, 6.18; N, 3.19.

Ethyl 4-(4-(*tert*-butyl)phenyl)-5,7-dimethoxy-3-(4-methoxyphenyl)quinoline-1(2*H*)-carboxylate (**9d**)

White solid (0.8 g, 57%). M.p. 118–120 °C; UV (MeOH): λ_max_ 212 (ε 67,271 cm^−1^M^−1^), 245 (22,589), 325 (15,303) nm; IR (KBr): ν_max_ 2936, 1693, 1606, 1513, 1451, 1329, 1248, 1202, 1168, 1138, 1032, 858, 831, 811, 760, 656 cm^−1^; ^1^H NMR (300 MHz, CDCl_3_): δ 1.27 (s, 9H, 3 × Me), 1.28 (t, *J* = 7.2 Hz, 3H, CH_3_CH_2_), 3.19 (s, 3H, OMe), 3.74 (s, 3H, OMe), 3.83 (s, 3H, OMe), 4.24 (q, *J* = 7.2 Hz, 2H, CH_2_CH_3_), 4.54 (s, 2H, CH_2_N), 6.18 (d, *J* = 3.0 Hz, 1H, ArH6), 6.65 (d, *J* = 8.7 Hz, 2H, ArH3′, ArH5′), 6.87 (d, *J* = 6.3 Hz, 2H, ArH3″, ArH5″), 6.90 (d, *J* = 5.7 Hz, 2H, ArH2″, ArH6″), 6.99 (d, *J* = 3.0 Hz, 1H, ArH8), 7.13 (d, *J* = 8.3 Hz, 2H, ArH2′, ArH6′); ^13^C NMR (75.6 MHz, CDCl_3_): δ 14.6 (Me), 31.4 (3 × MeC), 34.4 (CMe), 49.0 (CH_2_N), 55.1 (OMe), 55.4 (OMe), 55.5 (OMe), 62.1 (OCH_2_), 96.9 (ArC6, ArC8), 100.4 (ArC), 113.0 (ArC3′, ArC5′), 114.7 (CHAr′), 124.0 (ArC3″, ArC5″), 128.9 (ArC4), 129.8 (ArC2′, ArC6′), 131.8 (ArC1′), 132.5 (ArC2″, ArC6″), 137.7 (ArC1″), 139.8 (ArC), 148.5 (ArC4″), 153.7 (COO), 157.7 (ArC4′) 157.8 (ArC7), 159.5 (ArC5); HRMS (ESI) *m*/*z* Calcd. for C_31_H_36_NO_5_ (M + H)^+^: 502.2593. Found: 502.2579.

Ethyl 5,7-dimethoxy-3-(4-methoxyphenyl)-4-(*p*-tolyl)quinoline-1(2*H*)-carboxylate (**9e**)

White solid (0.8 g, 60.5%). M.p. 124–126 °C; UV (MeOH): λ_max_ 214 (ε 41,875 cm^−1^M^−1^), 248 (17,296), 326 (11,834) nm; IR (KBr): ν_max_ 2957, 2837, 1702, 1599, 1507, 1443, 1241, 1148, 1109, 1051, 1021, 824, 806, 775, 762, 720 cm^−1^; ^1^H NMR (300 MHz, CDCl_3_): δ 1.30 (t, *J* = 6.0 Hz, 3H, CH_3_CH_2_), 2.30 (s, 3H, MeAr″), 3.26 (s, 3H, OMe), 3.76 (s, 3H, OMe), 3.85 (s, 3H, OMe), 4.26 (q, *J* = 6.0 Hz, 2H, CH_2_CH_3_), 4.55 (s, 2H, CH_2_N), 6.21 (d, *J* = 3.0 Hz, 1H, ArH6), 6.69 (d, *J* = 9.0 Hz, 2H, ArH3′, ArH5′), 6.87–6.92 (m, 4H, ArH2″, ArH3″, ArH5″, ArH6″), 6.95 (d, *J* = 9.0 Hz, 2H, ArH2′, ArH6′), 7.01 (d, *J* = 3.0 Hz, 1H, ArH8); ^13^C NMR (75.6 MHz, CDCl_3_): δ 14.5 (Me), 21.2 (MeAr″), 49.0 (CH_2_N), 55.1 (OMe), 55.4 (OMe), 55.5 (OMe), 62.0 (OCH_2_), 96.8 (ArC6, ArC8), 100.4 (ArC), 113.1 (ArC3′, ArC5′), 114.6 (CHAr′), 127.9 (ArC3″, ArC5″), 129.1 (ArC4′), 129.7 (ArC2′, ArC6′), 131.8 (ArC1′), 132.5 (ArC2″, ArC6″), 135.0 (ArC1″), 137.5 (ArC), 139.8 (ArC4″), 153.6 (COO), 157.7 (ArC4′), 157.8 (ArC7), 159.5 (ArC5); HRMS (ESI) *m*/*z* Calcd. for C_28_H_29_NO_5_Na (M + Na)^+^: 482.1943. Found: 482.1930; Anal. Calcd. for C_28_H_29_NO_5_: C, 73.18; H, 6.36; N, 3.05. Found: C, 73.29; H, 6.47; N, 3.01.

Ethyl 5,7-dimethoxy-3,4-diphenylquinoline-1(2*H*)-carboxylate (**9f**)

White solid (0.8 g, 58%). M.p. 112–114 °C; UV (MeOH): λ_max_ 216 (ε 65,759 cm^−1^M^−1^), 248 (27,210), 324 (15,873) nm; IR (KBr): ν_max_ 2973, 2840, 1698, 1595, 1567, 1443, 1396, 1323, 1259, 1146, 1133, 1049, 1031, 1014, 885, 848, 832, 765, 699 cm^−1^; ^1^H NMR (300 MHz, CDCl_3_): δ 1.32 (t, *J* = 6.0 Hz, 3H, CH_3_CH_2_), 3.26 (s, 3H, OMe), 3.88 (s, 3H, OMe), 4.28 (q, *J* = 7.2 Hz, 2H, CH_2_CH_3_), 4.61 (s, 2H, CH_2_N), 6.23 (d, *J* = 3.0 Hz, 1H, ArH6), 6.96–7.04 (m, 5H, ArH′, Ar″H), 7.11–7.19 (m, 6H, ArH′, ArH″, ArH8); ^13^C NMR (75.6 MHz, CDCl_3_): δ 14.5 (Me), 48.9 (CH_2_N), 55.4 (2 × OMe), 62.1 (OCH_2_), 96.7 (ArC6, ArC8), 100.4 (ArC), 114.2 (CHAr′), 125.7 (ArC6′), 126.3 (ArC2′), 127.1 (ArC4′, ArC4″), 127.7 (ArC2″, ArC6″), 128.5 (ArC3′, ArC5′), 129.3 (ArC3″, ArC5″), 132.7 (ArC4), 139.8 (ArC), 139.9 (ArC1′), 140.5 (ArC1″), 153.6 (COO), 157.7 (ArC7), 159.8 (ArC5); HRMS (ESI) *m*/*z* Calcd. for C_26_H_25_NO_4_Na (M + Na)^+^: 438.1681. Found: 438.1667; Anal. Calcd. for C_26_H_25_NO_4_: C, 75.16; H, 6.06; N, 3.37. Found: C, 75.19; H, 6.16; N, 3.33.

Ethyl 5,7-dimethoxy-3-phenyl-4-(*p*-tolyl)quinoline-1(2*H*)-carboxylate (**9g**)

White solid (0.7 g, 53%). M.p. 118–120 °C; UV (MeOH): λ_max_ 203 (ε 100,357 cm^−1^M^−1^), 252 (54,057), 317 (30,190) nm; IR (KBr): ν_max_ 2938, 1708, 1599, 1574, 1443, 1397, 1320, 1265, 1203, 1151, 1136, 1047, 1034, 968, 939, 899, 808, 762, 699 cm^−1^; ^1^H NMR (300 MHz, CDCl_3_): δ 1.28 (t, *J* = 6.0 Hz, 3H, CH_3_CH_2_), 2.28 (s, 3H, MeAr″), 3.23 (s, 3H, OMe), 3.85 (s, 3H, OMe), 4.24 (q, *J* = 7.2 Hz, 2H, CH_2_CH_3_), 4.55 (s, 2H, CH_2_N), 6.20 (d, *J* = 3.0 Hz, 1H, ArH6), 6.82–7.00 (m, 7H, ArH′, ArH″), 7.08–7.11 (m, 3H, ArH′, ArH″, ArH8′); ^13^C NMR (75.6 MHz, CDCl_3_): δ 14.5 (Me), 21.2 (MeAr″), 48.9 (CH_2_N), 55.4 (OMe), 55.5 (OMe), 62.1 (OCH_2_), 96.8 (ArC6, ArC8), 114.4 (ArC), 126.2 (CHAr′), 127.7 (ArC6′), 127.8 (ArC2′), 127.9 (ArC4′), 128.0 (ArC5′), 128.3 (ArC3′), 128.5 (ArC5″), 129.1 (ArC3″), 132.5 (ArC4), 135.1 (ArC2″, ArC6″), 137.3 (ArC, ArC4″), 140.0 (ArC1″), 140.2 (ArC1′), 153.6 (COO), 157.8 (ArC7), 159.7 (ArC5); HRMS (ESI) *m*/*z* Calcd. for C_27_H_27_NO_4_Na (M + Na)^+^: 452.1838. Found: 452.1828; Anal. Calcd. for C_27_H_27_NO_4_: C, 75.50; H, 6.34; N, 3.26. Found: C, 75.56; H, 6.51; N, 3.14.

Ethyl *cis*-5,7-dimethoxy-3,4-bis(4-methoxyphenyl)-3,4-dihydroquinoline-1(2*H*)-carboxylate (**10b**)

White solid (0.6 g, 92%). M.p. 122–124 °C; UV (MeOH): λ_max_ 216 (ε 454,959 cm^−1^M^−1^), 284 (31,847), 336 (50,045) nm; IR (KBr): ν_max_ 2971, 2905, 2841, 1703, 1607, 1584, 1511, 1375, 1239, 1169, 1154, 1039, 1023, 936, 889, 811, 763 cm^−1^; ^1^H NMR (300 MHz, CDCl_3_): δ 1.33 (t, *J* = 9.0 Hz, 3H, CH_3_CH_2_), 3.31 (dt, *J* = 3.0, 12.0 Hz, 1H, H3), 3.60 (s, 3H, OMe), 3.61–3.70 (m, 1H, H2a), 3.72 (s, 3H, OMe), 3.79 (s, 3H, OMe), 3.85 (s, 3H, OMe), 4.06 (ddd, *J* = 3.0, 3.0, 12.0 Hz, 1H, H2b), 4.29 (q, *J* = 9.0 Hz, 2H, CH_2_CH_3_), 4.42 (dd, *J* = 3.0, 6.0 Hz, 1H, H4), 6.22 (d, *J* = 3.0 Hz, 1H, ArH6), 6.48 (d, *J* = 9.0 Hz, 2H, ArH3′, ArH5′), 6.59 (d, *J* = 9.0 Hz, 2H, ArH3″, ArH5″), 6.70–6.77 (m, 4H, ArH2′, ArH6′, ArH2″, ArH6″), 7.44 (d, *J* = 3.0 Hz, 1H, ArH8); ^13^C NMR (75.6 MHz, CDCl_3_): δ 14.5 (Me), 42.8 (ArC4), 42.9 (ArC3), 44.8 (CH_2_N), 55.0 (OMe), 55.1 (OMe), 55.3 (OMe), 55.6 (OMe), 61.9 (OCH_2_), 94.4 (ArC6, ArC8), 99.2 (ArC), 112.5 (ArC3′, ArC5′), 113.2 (ArC3″, ArC5″), 129.2 (ArC2′, ArC6′), 130.4 (ArC2″, ArC6″), 131.6 (ArC), 132.2 (ArC1″), 138.8 (ArC1′), 154.9 (COO), 156.9 (ArC5), 157.8 (ArC5), 158.2 (ArC4′), 158.7 (ArC4″); HRMS (ESI) *m*/*z* Calcd. for C_28_H_31_NO_6_Na (M + Na)^+^: 500.2049. Found: 500.2034; Anal. Calcd. for C_28_H_31_NO_6_: C, 70.42; H, 6.54; N, 2.93. Found: C, 70.58; H, 6.72; N, 2.88.

Ethyl *cis*-5,7-dimethoxy-3-(4-methoxyphenyl)-4-phenyl-3,4-dihydroquinoline-1(2*H*)-carboxylate (**10c**)

White solid (0.6 g, 95%). M.p. 160–162 °C; UV (MeOH): λ_max_ 216 (ε 54,328 cm^−1^M^−1^), 283 (2388), 336 (5970) nm; IR (KBr): ν_max_ 3001, 2977, 2950, 2837, 1710, 1590, 1516, 1251, 1239, 1060, 1043, 830, 758, 701 cm^−1^; ^1^H NMR (300 MHz, CDCl_3_): δ 1.33 (t, *J* = 6.0 Hz, 3H, CH_3_CH_2_), 3.36 (dt, *J* = 3.0, 12.0 Hz, 1H, H3), 3.58 (s, 3H, OMe), 3.67 (t, *J* = 12.0 Hz, 1H, H2a), 3.78 (s, 3H, OMe), 3.85 (s, 3H, OMe), 4.09 (ddd, *J* = 3.0, 6.0, 12.0 Hz, 1H, H2b), 4.29 (q, *J* = 6.0 Hz, 2H, CH_2_CH_3_), 4.45 (d, *J* = 3.0, 1H, H4), 6.21 (d, *J* = 3.0 Hz, 1H, ArH6), 6.56–6.59 (m, 2H, Ph), 6.68–6.75 (m, 4H, ArH2′, ArH3′, ArH5′, ArH6′), 7.01–7.10 (m, 3H, Ph), 7.45 (d, *J* = 3.0 Hz, 1H, ArH8); ^13^C NMR (75.6 MHz, CDCl_3_): δ 14.5 (Me), 42.9 (ArC4), 43.7 (ArC3), 44.8 (CH_2_N), 55.1 (OMe), 55.3 (OMe), 55.6 (OMe), 61.9 (OCH_2_), 94.4 (ArC6, ArC8), 99.2 (ArC), 113.2 (ArC3′, ArC5′), 125.9 (ArC4″), 127.1 (ArC2′, Ar′C6), 129.1 (ArC2″, ArC6″), 129.5 (ArC3″, ArC5″), 132.1 (ArC), 138.9 (ArC1″), 139.7 (ArC1′), 154.9 (COO), 157.0 (ArC5), 158.3 (ArC5), 158.8 (ArC4′); HRMS (ESI) *m*/*z* Calcd. for C_27_H_29_NO_5_Na (M + Na)^+^: 470.1943. Found: 470.1927; Anal. Calcd. for C_27_H_29_NO_5_: C, 72.46; H, 6.53; N, 3.13. Found: C, 72.65; H, 6.45; N, 3.19.

Ethyl *cis*-4-(4-(*tert*-butyl)phenyl)-5,7-dimethoxy-3-(4-methoxyphenyl)-3,4-dihydroquinoline-1(2*H*)-carboxylate (**10d**)

White solid (0.65 g, 91.5%). M.p. 116–118 °C; UV (MeOH): λ_max_ 203 (ε 71,732 cm^−1^M^−1^), 220 (74,830) nm; IR (KBr): ν_max_ 3154, 2959, 1712, 1590, 1512, 1373, 1267, 1240, 1060, 1042, 838, 812, 762 cm^−1^; ^1^H NMR (300 MHz, CDCl_3_): δ 1.28 (s, 9H, 3 × Me), 1.38 (t, *J* = 6.0 Hz, 3H, CH_3_CH_2_), 3.36 (dt, *J* = 6.0, 12.0 Hz, 1H, H3), 3.64 (s, 3H, OMe), 3.72 (t, *J* = 15.0 Hz, 1H, H2a), 3.82 (s, 3H, OMe), 3.89 (s, 3H, OMe), 4.08 (ddd, *J* = 3.0, 6.0, 12.0 Hz, 1H, H2b), 4.34 (q, *J* = 6.0 Hz, 2H, CH_2_CH_3_), 4.48 (d, *J* = 6.0, 1H, H4), 6.26 (d, *J* = 3.0 Hz, 1H, ArH6), 6.54 (d, *J* = 9.0 Hz, 2H, ArH3′, ArH5′), 6.71–6.78 (m, 4H, ArH2′, ArH6′, ArH2″, ArH6″), 7.09 (d, *J* = 9H, 2H, ArH3″, ArH5″), 7.47 (d, *J* = 3.0 Hz, 1H, ArH8); ^13^C NMR (75.6 MHz, CDCl_3_): δ 14.6 (Me), 31.4 (3 × MeC), 34.3 (CMe), 43.2 (ArC4, ArC3), 45.2 (CH_2_N), 55.2 (OMe), 55.4 (OMe), 55.7 (OMe), 62.0 (OCH_2_), 94.5 (ArC6, ArC8), 99.5 (ArC), 113.2 (ArC3′, ArC5′), 124.1 (ArC3″, ArC5″), 129.2 (ArC2′, ArC6′), 129.3 (ArC2″, ArC6″), 132.3 (ArC), 136.3 (ArC1″), 138.9 (ArC1′), 148.8 (ArC4″), 155.1 (COO), 157.0 (ArC5), 158.4 (ArC7), 158.8 (ArC4′); HRMS (ESI) *m*/*z* Calcd. for C_31_H_37_NO_5_Na (M + Na)^+^: 526.2569. Found: 526.2555; Anal. Calcd. for C_31_H_37_NO_5_: C, 73.93; H, 7.41; N, 2.78. Found: C, 73.65; H, 7.45; N, 2.74.

Ethyl *cis*-5,7-dimethoxy-3-(4-methoxyphenyl)-4-(*p*-tolyl)-3,4-dihydroquinoline-1(2*H*)-carboxylate (**10e**)

White solid (0.65 g, 95.5%). M.p. 140–142 °C; UV (MeOH): λ_max_ 217 (ε 61,337 cm^−1^M^−1^), 283 (2067), 336 (7581) nm; IR (KBr): ν_max_ 2978, 1704, 1586, 1513, 1376, 1241, 1169, 1058, 1042, 1024, 937, 889, 809, 761 cm^−1^; ^1^H NMR (300 MHz, CDCl_3_): δ 1.36 (t, *J* = 7.2 Hz, 3H, CH_3_CH_2_), 2.26 (s, 3H, MeAr″), 3.35 (dt, *J* = 4.2, 13.5 Hz, 1H, H3), 3.62 (s, 3H, OMe), 3.69 (t, *J* = 13.5 Hz, 1H, H2a), 3.81 (s, 3H, OMe), 3.87 (s, 3H, OMe), 4.08 (ddd, *J* = 1.5, 4.50, 12.3 Hz, 1H, H2b), 4.31 (q, *J* = 7.2 Hz, 2H, CH_2_CH_3_), 4.46 (d, *J* = 3.6 Hz, 1H, H4), 6.23 (d, *J* = 2.4 Hz, 1H, ArH6), 6.47 (d, *J* = 8.1 Hz, 2H, ArH3′, ArH5′), 6.76 (s, 4H, ArH2″, ArH3″, ArH5″, ArH6″), 6.86 (d, *J* = 7.8 Hz, 2H, ArH2′, ArH6′), 7.47 (d, *J* = 2.1 Hz, 1H, ArH8); ^13^C NMR (75.6 MHz, CDCl_3_): δ 14.6 (Me), 21.0 (MeAr″), 43.0 (ArC4), 43.3 (ArC3), 45.0 (CH_2_N), 55.2 (OMe), 55.4 (OMe), 55.7 (OMe), 62.0 (OCH_2_), 94.5 (ArC6, ArC8), 99.3 (ArC), 113.3 (ArC3′, ArC5′), 128.0 (ArC2′, ArC6′), 129.3 (ArC2″, ArC6″), 129.5 (ArC3″, ArC5″), 132.2 (ArC), 135.5 (ArC4″), 136.4 (ArC1′), 138.9 (ArC1″), 155.0 (COO), 157.0 (ArC5), 158.4 (ArC7), 158.8 (ArC4′); HRMS (ESI) *m*/*z* Calcd. for C_28_H_31_NO_5_Na (M + Na)^+^: 484.2100. Found: 484.2087; Anal. Calcd. for C_28_H_31_NO_5_: C, 72.86; H, 6.77; N, 3.03. Found: C, 72.89; H, 6.93; N, 2.98.

Ethyl *cis*-5,7-dimethoxy-3,4-diphenyl-3,4-dihydroquinoline-1(2*H*)-carboxylate (**10f**)

White solid (0.65 g, 93%). M.p. 90–92 °C; UV (MeOH): λ_max_ 215 (ε 49,140 cm^−1^M^−1^), 247 (20,147), 337 (10,319) nm; IR (KBr): ν_max_ 3001, 2939, 2839, 1687, 1579, 1489, 1454, 1368, 1307, 1214, 1202, 1173, 1063, 1039, 1022, 949, 882, 836, 823, 758, 656 cm^−1^; ^1^H NMR (300 MHz, CDCl_3_): δ 1.37 (t, *J* = 7.2 Hz, 3H, CH_3_CH_2_), 3.46 (dt, *J* = 4.2, 13.2 Hz, 1H, H3), 3.61 (s, 3H, OMe), 3.77 (t, *J* = 12.9 Hz, 1H, H2a), 3.89 (s, 3H, OMe), 4.20 (ddd, *J* = 1.5, 4.2, 12.6 Hz, 1H, H2b), 4.33 (q, *J* = 7.2 Hz, 2H, CH_2_CH_3_), 4.55 (d, *J* = 4.8 Hz, 1H, H4), 6.26 (d, *J* = 2.4 Hz, 1H, ArH6), 6.57–6.61 (m, 2H, ArH′), 6.82–6.85 (m, 2H, ArH″), 7.03–7.14 (m, 3H, ArH′), 7.21–7.25 (m, 3H, ArH″), 7.50 (d, *J* = 2.4 Hz, 1H, ArH8); ^13^C NMR (75.6 MHz, CDCl_3_): δ 14.6 (Me), 43.7 (ArC4), 43.9 (ArC3), 44.5 (CH_2_N), 55.4 (OMe), 55.7 (OMe), 62.0 (OCH_2_), 94.6 (ArC6), 99.3 (ArC8), 113.5 (ArC), 126.0 (ArC4′), 126.8 (ArC4″), 127.2 (ArC2″, ArC6″), 127.9 (ArC3′, ArC5′), 128.2 (ArC2′, ArC6′), 129.6 (ArC3″, ArC5″), 139.1 (ArC), 139.9 (ArC1″), 140.1 (ArC1′), 155.0 (COO), 157.2 (ArC5), 159.0 (ArC7); HRMS (ESI) *m*/*z* Calcd. for C_26_H_27_NO_4_Na (M + Na)^+^: 440.1838. Found: 440.1822; Anal. Calcd. for C_26_H_27_NO_4_: C, 74.80; H, 6.52; N, 3.35. Found: C, 74.96; H, 6.66; N, 3.32.

Ethyl *cis*-5,7-dimethoxy-3-phenyl-4-(*p*-tolyl)-3,4-dihydroquinoline-1(2*H*)-carboxylate (**10g**)

White solid (0.6 g, 95%). M.p. 122–124 °C; UV (MeOH): λ_max_ 216 (ε 134,352 cm^−1^M^−1^) nm; IR (KBr): ν_max_ 2939, 1723, 1690, 1589, 1455, 1373, 1325, 1287, 1243, 1170, 1140, 1057, 1044, 940, 885, 821, 808, 761, 698 cm^−1^; ^1^H NMR (300 MHz, CDCl_3_): δ 1.33 (t, *J* = 6.9 Hz, 3H, CH_3_CH_2_), 2.23 (s, 3H, MeAr″), 3.38 (dt, *J* = 4.2, 13.2 Hz, 1H, H3), 3.59 (s, 3H, OMe), 3.72 (t, *J* = 13.2 Hz, 1H, H2a), 3.85 (s, 3H, OMe), 4.12 (ddd, *J* = 1.5, 4.2, 12.3 Hz, 1H, H2b), 4.29 (q, *J* = 6.9 Hz, 2H, CH_2_CH_3_), 4.48 (d, *J* = 4.2 Hz, 1H, H4), 6.21 (d, *J* = 2.4 Hz, 1H, ArH6), 6.43 (d, *J* = 8.1 Hz, 2H, ArH3″, ArH5″), 6.81–6.84 (m, 4H, Ph), 7.18–7.21 (m, 3H, ArH2″, ArH6″, Ph), 7.45 (d, *J* = 2.4 Hz, 1H, ArH8); ^13^C NMR (75.6 MHz, CDCl_3_): δ 14.6 (Me), 21.0 (MeAr″), 43.3 (ArC4), 43.7 (ArC3), 44.7 (CH_2_N), 55.4 (OMe), 55.7 (OMe), 62.0 (OCH_2_), 94.5 (ArC6), 99.3 (ArC8), 113.9 (ArC), 126.7 (ArC4′), 127.9 (ArC2′, ArC6′), 128.0 (Ar″C2, Ar″C6), 128.3 (ArC3′, ArC5′), 129.4 (ArC3″, ArC5″), 135.5 (ArC4″), 136.4 (ArC), 139.0 (ArC1″), 140.2 (ArC1′), 155.0 (COO), 157.1 (ArC5), 158.9 (ArC7); HRMS (ESI) *m*/*z* Calcd. for C_27_H_29_NO_4_Na (M + Na)^+^: 454.1994. Found: 454.1980; Anal. Calcd. for C_27_H_29_NO_4_: C, 75.15; H, 6.77; N, 3.25. Found: C, 75.40; H, 6.87; N, 3.22.

*cis*-5,7-Dimethoxy-3,4-bis(4-methoxyphenyl)-1,2,3,4-tetrahydroquinoline (**3b**)

White solid (0.35 g, 82%). M.p. 102–104 °C; UV (MeOH): λ_max_ 214 (ε 62,383 cm^−1^M^−1^), 246 (23,705), 337 (13,100) nm; IR (KBr): ν_max_ 2935, 1703, 1608, 1583, 1511, 1234, 1204, 1168, 1143, 1032, 953, 826, 763 cm^−1^; ^1^H NMR (300 MHz, CDCl_3_): δ 3.21 (ddd, *J* = 3.0, 6.0, 9.0 Hz, 1H, H2b), 3.31 (dt, *J* = 6.0, 12.0 Hz, 1H, H3), 3.53–3.60 (m, 1H, H2a), 3.57 (s, 3H, OMe), 3.72 (s, 3H, OMe), 3.77 (s, 3H, OMe), 3.78 (s, 3H, OMe), 4.13 (bs, 1H, NH), 4.31 (dd, *J* = 3.0, 6.0 Hz, 1H, H4), 5.82 (d, *J* = 3.0 Hz, 1H, ArH6), 5.84 (d, *J* = 3.0 Hz, 1H, ArH8), 6.54–6.58 (m, 4H, ArH3′, ArH5′, ArH3″, ArH5″), 6.63 (d, *J* = 9.0 Hz, 2H, ArH2″, ArH6″), 6.70 (d, *J* = 9.0 Hz, 2H, ArH2′, ArH6′); ^13^C NMR (75.6 MHz, CDCl_3_): δ 40.9 (ArC4), 42.0 (ArC3), 42.4 (CH_2_N), 54.9 (OMe), 55.0 (OMe), 55.1 (OMe), 55.4 (OMe), 87.9 (ArC6), 90.7 (ArC8), 105.5 (ArC), 112.1 (ArC3′, ArC5′), 113.0 (ArC3″, ArC5″), 129.0 (ArC2′, ArC6′), 130.6 (ArC2″, ArC6″), 133.4 (ArC1″), 134.3 (ArC1′), 145.0 (ArC), 157.4 (ArC5), 157.9 (ArC7), 158.3 (ArC4′), 159.9 (ArC1″); HRMS (ESI) *m*/*z* Calcd. for C_25_H_28_NO_4_ (M + H)^+^: 406.2018. Found: 406.2006; Anal. Calcd. for C_25_H_27_NO_4_.0.25 H_2_O: C, 73.24; H, 6.76; N, 3.42. Found: C, 73.48; H, 6.82; N, 3.38.

*cis*-5,7-Dimethoxy-3-(4-methoxyphenyl)-4-phenyl-1,2,3,4-tetrahydroquinoline (**3c**)

White solid (0.4 g, 87%). M.p. 166–168 °C; UV (MeOH): λ_max_ 203 (ε 51,440 cm^−1^M^−1^), 216 (51,440), 337 (5144) nm; IR (KBr): ν_max_ 3425, 3006, 2964, 2906, 2838, 2877, 1603, 1503, 1402, 1207, 1111, 1134, 1090, 1054, 810, 796, 728 cm^−1^; ^1^H NMR (300 MHz, CDCl_3_): δ 3.21 (ddd, *J* = 3.0, 3.0, 9.0 Hz, 1H, H2b), 3.35 (dt, *J* = 3.0, 12.0 Hz, 1H, H3), 3.56 (s, 3H, OMe), 3.59–3.64 (m, 1H, H2a), 3.76 (s, 3H, OMe), 3.78 (s, 3H, OMe), 4.14 (bs, 1H, NH), 4.35 (d, *J* = 3.0, 1H, H4), 5.82 (d, *J* = 3.0 Hz, 1H, ArH6), 5.85 (d, *J* = 3.0 Hz, 1H, ArH8), 6.60–6.71 (m, 6H, ArH3′, ArH5′, ArH2″, ArH4″, ArH5″, ArH6″), 7.01–7.06 (m, 3H, ArH2′, ArH6′, ArH3″); ^13^C NMR (75.6 MHz, CDCl_3_): δ 40.9 (ArC4), 41.9 (ArC3), 43.3 (CH_2_N), 55.0 (OMe), 55.1 (OMe), 55.3 (OMe), 87.9 (ArC6), 90.6 (ArC8), 105.2 (ArC), 113.0 (ArC3′, ArC5′), 125.5 (ArC4″), 126.7 (ArC2′, ArC6′), 128.9 (ArC2″, ArC6″), 129.8 (ArC3″, ArC5″), 133.3 (ArC), 142.1 (ArC1″), 145.1 (ArC1′), 157.9 (ArC5), 158.4 (ArC7), 159.9 (ArC4′); HRMS (ESI) *m*/*z* Calcd. for C_24_H_26_NO_3_ (M + H)^+^: 376.1913. Found: 376.1902; Anal. Calcd. for C_24_H_25_NO_3_: C, 76.77; H, 6.71; N, 3.73. Found: C, 77.00; H, 6.98; N, 3.70.

*cis*-4-(4-(*tert*-Butyl)phenyl)-5,7-dimethoxy-3-(4-methoxyphenyl)-1,2,3,4-tetrahydroquinoline (**3d**)

White solid (0.4 g, 90.5%). M.p. 118–120 °C; UV (MeOH): λ_max_ 203 (ε 85,611 cm^−1^M^−1^), 216 (80,755) nm; IR (KBr): ν_max_ 3427, 2960, 1609, 1509, 1222, 1211, 1133, 1109, 1050, 1023, 831, 805, 704 cm^−1^; ^1^H NMR (300 MHz, CDCl_3_): δ 1.23 (s, 9H, 3 × Me), 3.24 (ddd, *J* = 3.0, 6.0, 15.0 Hz, 1H, H2b), 3.35 (dt, *J* = 3.0, 15.0 Hz, 1H, H3), 3.56–3.63 (m, 1H, H2a), 3.57 (s, 3H, OMe), 3.76 (s, 3H, OMe), 3.77 (s, 3H, OMe), 3.84 (bs, 1H, NH), 4.32 (d, *J* = 3.0, 1H, H4), 5.89 (d, *J* = 3.0 Hz, 1H, ArH6), 5.97 (d, *J* = 3.0 Hz, 1H, ArH8), 6.53–6.57 (m, 4H, ArH3′, ArH5′, ArH2″, ArH4″), 6.65 (d, *J* = 9.0 Hz, 2H, ArH2′, ArH6′), 7.03 (d, *J* = 9.0 Hz, 2H, ArH3″, ArH5″); ^13^C NMR (75.6 MHz, CDCl_3_): δ 31.4 (3 × Me), 34.2 (CMe), 41.2 (ArC4, ArC3), 42.7 (CH_2_N), 55.2 (2 × OMe), 55.5 (OMe), 89.5 (ArC6), 91.8 (ArC8), 106.9 (ArC), 113.0 (ArC3′, ArC5′), 123.7 (Ar″C3, Ar″C5), 129.1 (ArC2′, ArC6′), 129.3 (ArC2″, ArC6″), 133.3 (ArC), 138.6 (ArC1″, ArC1′), 148.4 (ArC4″), 158.1 (ArC5), 158.5 (ArC7), 160.0 (Ar′C4); HRMS (ESI) *m*/*z* Calcd. for C_25_H_24_NO_4_ (M + H)^+^: 402.1705. Found: 402.1692.

*cis*-5,7-Dimethoxy-3-(4-methoxyphenyl)-4-(*p*-tolyl)-1,2,3,4-tetrahydroquinoline (**3e**)

White solid (0.4 g, 87%). M.p. 170–172 °C; UV (MeOH): λ_max_ 203 (ε 54,535 cm^−1^M^−1^), 212 (55,648), 246 (21,702), 336 (11,686) nm; IR (KBr): ν_max_ 3418, 3002, 2918, 2837, 1613, 1511, 1223, 1110, 1099, 1049, 1030, 822, 767 cm^−1^; ^1^H NMR (300 MHz, CDCl_3_): δ 2.16 (s, 3H, MeAr″), 3.13 (ddd, *J* = 1.5, 3.6, 10.8 Hz, 1H, H2a), 3.25 (dt, *J* = 3.9, 12.3 Hz, 1H, H3), 3.41–3.54 (m, 1H, H2b), 3.49 (s, 3H, OMe), 3.69 (s, 3H, OMe), 3.70 (s, 3H, OMe), 4.05 (bs, 1H, NH), 4.26 (d, *J* = 3.6, 1H, H4), 5.74 (d, *J* = 2.4 Hz, 1H, ArH6), 5.77 (d, *J* = 2.1 Hz, 1H, ArH8), 6.46 (d, *J* = 8.1 Hz, 2H, ArH3′, ArH5′), 6.55 (d, *J* = 8.7 Hz, 2H, ArH2″, ArH6″), 6.63 (d, *J* = 9.0 Hz, 2H, ArH3″, ArH5″), 6.77 (d, *J* = 8.4 Hz, 2H, ArH2′, ArH6′); ^13^C NMR (75.6 MHz, CDCl_3_): δ 21.0 (MeAr″), 41.1 (ArC4), 42.0 (ArC3), 42.9 (CH_2_N), 55.1 (OMe), 55.2 (OMe), 55.5 (OMe), 88.0 (ArC6), 90.8 (ArC8), 105.6 (ArC), 113.1 (ArC3′, ArC5′), 127.6 (ArC2′, ArC6′), 129.1 (ArC2″, ArC6″), 129.8 (ArC3″, ArC5″), 133.6 (ArC), 134.9 (ArC4″), 139.0 (ArC1″), 145.1 (ArC1′), 158.0 (ArC5), 158.5 (ArC7), 160.0 (ArC4′); HRMS (ESI) *m*/*z* Calcd. for C_25_H_28_NO_3_ (M + H)^+^: 390.2069. Found: 390.2054; Anal. Calcd. for C_25_H_27_NO_3_: C, 77.09; H, 6.99; N, 3.60. Found: C, 76.97; H, 7.09; N, 3.54.

*cis*-5,7-Dimethoxy-3,4-diphenyl-1,2,3,4-tetrahydroquinoline (**3f**)

White solid (0.4 g, 86.5%). M.p. 180–182 °C; UV (MeOH): λ_max_ 214 (ε 43,196 cm^−1^M^−1^), 337 (6479) nm; IR (KBr): ν_max_ 3424, 2873, 1598, 1507, 1451, 1401, 1367, 1219, 1203, 1132, 1095, 1078, 1046, 936, 796, 764, 662 cm^−1^; ^1^H NMR (300 MHz, CDCl_3_): δ 3.27 (ddd, *J* = 1.5, 3.6, 11.1 Hz, 1H, H2a), 3.41 (dt, *J* = 3.9, 12.6 Hz, 1H, H3), 3.56 (s, 3H, OMe), 3.66 (t, *J* = 12.3 Hz, 1H, H2b), 3.78 (s, 3H, OMe), 4.05 (bs, 1H, NH), 4.41 (dd, *J* = 1.2, 4.8 Hz, 1H, H4), 5.83 (d, *J* = 2.4 Hz, 1H, ArH6), 5.87 (d, *J* = 2.1 Hz, 1H, ArH8), 6.62–6.65 (m, 2H, ArH′), 6.70–6.74 (m, 2H, ArH″), 6.99–7.07 (m, 3H, ArH′), 7.12–7.16 (m, 3H, ArH″); ^13^C NMR (75.6 MHz, CDCl_3_): δ 40.7 (ArC4), 42.8 (ArC3), 43.4 (CH_2_N), 55.1 (OMe), 55.4 (OMe), 88.2 (ArC6), 90.9 (ArC8), 105.4 (ArC), 125.7 (ArC4″), 126.3 (ArC4′), 126.8 (ArC2′, ArC6′), 127.7 (ArC2″, ArC6″), 128.1 (ArC3′, ArC5′), 129.8 (ArC3″, ArC5″), 141.3 (ArC), 142.2 (ArC1″), 145.0 (ArC1′), 158.5 (ArC5), 160.1 (ArC7); HRMS (ESI) *m*/*z* Calcd. for C_23_H_24_NO_2_ (M + H)^+^: 346.1807. Found: 346.1794; Anal. Calcd. for C_23_H_23_NO_2_: C, 79.97; H, 6.71; N, 4.05. Found: C, 80.00; H, 6.82; N, 4.02.

*cis*-5,7-Dimethoxy-3-phenyl-4-(*p*-tolyl)-1,2,3,4-tetrahydroquinoline (**3g**)

White solid (0.4 g, 91%). M.p. 184–186 °C; UV (MeOH): λ_max_ 216 (ε 110,601 cm^−1^M^−1^) nm; IR (KBr): ν_max_ 3395, 2850, 1617, 1589, 1514, 1470, 1351, 1315, 1222, 1170, 1130, 1096, 1046, 945, 755, 695 cm^−1^; ^1^H NMR (300 MHz, CDCl_3_): δ 2.23 (s, 3H, MeAr″), 3.26 (ddd, *J* = 1.8, 3.6, 11.1 Hz, 1H, H2a), 3.38 (dt, *J* = 3.9, 12.3 Hz, 1H, H3), 3.57 (s, 3H, OMe), 3.65 (t, *J* = 11.1 Hz, 1H, H2b), 3.78 (s, 3H, OMe), 4.39 (dd, *J* = 1.2, 4.2 Hz, 1H, H4), 5.83 (d, *J* = 2.1 Hz, 1H, ArH6), 5.86 (d, *J* = 2.1 Hz, 1H, ArH8), 6.51 (d, *J* = 8.1 Hz, 2H, ArH3″, ArH5″), 6.73–6.76 (m, 2H, Ph), 6.83 (d, *J* = 7.8 Hz, 2H, ArH2″, ArH6″), 7.15–7.17 (m, 3H, Ph); ^13^C NMR (75.6 MHz, CDCl_3_): δ 21.0 (MeAr″), 40.7 (ArC4), 42.8 (ArC3), 42.9 (CH_2_N), 55.1 (OMe), 55.5 (OMe), 88.2 (ArC6), 90.9 (ArC8), 105.6 (ArC), 126.3 (ArC4′), 127.6 (ArC2′, ArC6′), 127.7 (ArC2″, ArC6″), 128.2 (ArC3′, ArC5′), 129.7 (ArC3″, ArC5″), 135.0 (ArC4″), 139.0 (ArC), 142.5 (ArC1″), 145.0 (ArC1′), 158.5 (ArC5), 160.0 (ArC7); HRMS (ESI) *m*/*z* Calcd. for C_24_H_26_NO_2_ (M + H)^+^: 360.1964. Found: 360.1952; Anal. Calcd. for C_24_H_25_NO_2_: C, 80.19; H, 7.01; N, 3.90. Found: C, 80.00; H, 7.13; N, 3.85.

4-(4-(*tert*-Butyl)phenyl)-3-(4-hydroxyphenyl)-5-hydroxy-7-methoxy-1,2,3,4-tetrahydroquinoline (**13d**)

Yellow solid (0.06 g, 30%). M.p. 232–234 °C; UV (MeOH): λ_max_ 203 (ε 63,067 cm^−1^M^−1^), 216 (65,388) nm; IR (KBr): ν_max_ 3470, 3383, 2959, 2925, 2853, 1619, 1512, 1461, 1376, 1218, 1197, 1179, 1125, 1106, 1076, 1054, 825, 798, 726 cm^−1^; ^1^H NMR (300 MHz, Acetone-*d_6_*): δ 1.31 (s, 9H, 3 × Me), 3.22–3.36 (m, 2H, H2a, H3), 3.68–3.82 (m, 1H, H2b), 3.72 (s, 3H, OMe), 4.20 (dd, *J* = 0.9, 4.5 Hz, 1H, H4), 5.33 (bs, 1H, NH), 5.80 (d, *J* = 2.4 Hz, 1H, ArH6), 5.91 (d, *J* = 2.1 Hz, 1H, ArH8), 6.62–6.73 (m, 6H, ArH2′, ArH3′, ArH5′, ArH6′, ArH2″, ArH6″), 7.14 (d, *J* = 8.4 Hz, 2H, ArH3″, ArH5″), 7.71 (s, 1H, OH), 8.16 (s, 1H, OH); ^13^C NMR (75.6 MHz, CDCl_3_): δ 30.9 (3 × MeC), 33.9 (CMe), 40.9 (ArC4), 43.1 (ArC3), 43.2 (CH_2_N), 54.1 (OMe), 90.2 (ArC6, ArC8), 104.1 (ArC), 114.6 (ArC3′, ArC5′), 123.4 (ArC3″, ArC5″), 128.9 (ArC2″, ArC6″), 129.6 (ArC2′, ArC6′), 139.8 (ArC1″), 139.8 (ArC1′), 147.9 (ArC), 148.0 (ArC4″), 155.5 (ArC4′), 158.2 (ArC5), 160.1 (ArC7); HRMS (ESI) *m*/*z* Calcd. for C_26_H_30_NO_3_ (M + H)^+^: 404.2226. Found: 404.2209.

5-Hydroxy-7-methoxy-3,4-diphenyl-1,2,3,4-tetrahydroquinoline (**13f**)

Yellow solid (0.07 g, 38.5%). M.p. 198–200 °C; UV (MeOH): λ_max_ 216 (ε 53,867 cm^−1^M^−1^), 246 (14,304), 337 (7152) nm; IR (KBr): ν_max_ 3675, 3377, 2971, 2901, 1625, 1597, 1490, 1450, 1410, 1375, 1231, 1212, 1153, 1118, 1080, 1050, 798, 764, 662 cm^−1^; ^1^H NMR (300 MHz, Acetone-*d_6_*): δ 3.24–3.31 (m, 1H, H2a), 3.38 (dt, *J* = 3.9, 12.3 Hz, 1H, H3), 3.62–3.80 (m, 1H, H2b), 3.69 (s, 3H, OMe), 4.45 (dd, *J* = 1.2, 4.8 Hz, 1H, H4), 5.39 (bs, 1H, NH), 5.76 (d, *J* = 2.4 Hz, 1H, ArH6), 5.89 (d, *J* = 2.1 Hz, 1H ArH8), 6.67–6.70 (m, 2H, ArH′), 6.79–6.82 (m, 2H, ArH″), 6.99–7.06 (m, 3H, ArH′), 7.14–7.18 (m, 3H, ArH″), 7.78 (s, 1H, OH); ^13^C NMR (75.6 MHz, Acetone-*d_6_*): δ 40.3 (ArC4), 42.7 (ArC3), 43.7 (CH_2_N), 54.1 (OMe), 90.1 (ArC6), 90.2 (ArC8), 103.6 (ArC), 125.4 (ArC4″), 126.1 (ArC4′), 126.6 (ArC2′, ArC6′), 127.6 (ArC2″, ArC6″), 128.0 (ArC3′, ArC5′), 130.0 (ArC3″, ArC5″), 141.9 (ArC), 142.7 (ArC1″), 146.3 (ArC1′), 155.6 (ArC5), 160.0 (ArC7); HRMS (ESI) *m*/*z* Calcd. for C_22_H_22_NO_2_ (M + H)^+^: 332.1951. Found: 332.1636.

5-Hydroxy-7-methoxy-3-phenyl-4-(*p*-tolyl)-1,2,3,4-tetrahydroquinoline (**13g**)

Yellow solid (0.06 g, 32%). M.p. 128–130 °C; UV (MeOH): λ_max_ 203 (ε 25,755 cm^−1^M^−1^) nm; IR (KBr): ν_max_ 2922, 2852, 1610, 1510, 1451, 1366, 1330, 1286, 1238, 1202, 1111, 1028, 826, 802, 722, 697 cm^−1^; ^1^H NMR (300 MHz, CDCl_3_): δ 2.22 (s, 3H, MeAr″), 3.24 (ddd, *J* = 1.2, 3.3, 10.8 Hz, 1H, H2a), 3.43 (dt, *J* = 4.8, 12.3 Hz, 1H, H3), 3.63 (t, *J* = 12.3 Hz, 1H, H2b), 3.73 (s, 3H, OMe), 4.25 (d, *J* = 4.8 Hz, 1H, H4), 5.78 (d, *J* = 2.1 Hz, 1H, ArH6), 5.85 (d, *J* = 2.1 Hz, 1H, ArH8), 6.55 (d, *J* = 8.1 Hz, 2H, ArH3″, ArH5″), 6.71–6.74 (m, 2H, Ph), 6.84 (d, *J* = 7.80 Hz, 2H, ArH2″, ArH6″), 7.12–7.15 (m, 3H, Ph); ^13^C NMR (75.6 MHz, CDCl_3_): δ 21.0 (MeAr″), 40.6 (ArC4), 43.3 (ArC3), 43.5 (CH_2_N), 54.1 (OMe), 91.6 (ArC6), 91.9 (ArC8), 104.2 (ArC), 126.4 (ArC4′), 127.8 (ArC2′, ArC6′), 128.1 (ArC2″, ArC6″), 128.2 (ArC3′, ArC5′), 129.8 (ArC3″, ArC5″), 136.0 (ArC4″), 137.7 (ArC), 140.9 (ArC1″), 145.6 (Ar′C1), 154.7 (ArC5), 160.1 (ArC7); HRMS (ESI) *m*/*z* Calcd. for C_23_H_24_NO_2_ (M + H)^+^: 346.1807. Found: 346.1791.

## 4. Conclusions

Attempts to synthesize 3,4-disubstituted 1,2-dihydroquinolines by a Grignard reaction approach were unsuccessful and resulted in the formation of the more stable quinoline moiety. 

However, hydrogenation of *N*-protected 1,2-dihydroquinolines resulted in the formation of the 1,2,3,4-tetrahydroquinolines with *cis* configuration. Demethylation of these 5,7-dimethoxy tetrahydroquinolines in the presence of two equivalents of BBr_3_ for each methoxy group resulted in the formation of the corresponding hydroxyl compounds, though the compounds were seen to have a low stability. The use of one equivalent of BBr_3_ for each methoxy group resulted in the formation of 5-hydroxy analogues in good yields.

The biological activity of compound **3c** offers opportunities for further SAR studies on various C4 and aromatic substituents of our 1,2,3,4-tetrahydroquinoline scaffold, which could result in lead compounds with improved anticancer potency. Testing of the mono-deprotected analogues **13** would also provide insight into the role of the methoxy groups in the activity of our compounds.

## Figures and Tables

**Table 1:** Analogues of 3,4-diaryl-5,7-dimethoxy-1,2,3,4-tetrahydroquinolines.

Entry	R	Ar	Yield (%)
**3b**	4-OMe	4-OMeC_6_H_4_	82
**3c**	4-OMe	Ph	87
**3d**	4-OMe	4-^t^BuC_6_H_4_	90
**3e**	4-OMe	4-MeC_6_H_4_	87
**3f**	H	Ph	86
**3g**	H	4-MeC_6_H_4_	91

**Table 2:** Hydroxy analogues of tetrahydroquinolines.

Entry	R	Ar	Yield (%)
**13d**	OH	4-^t^BuC_6_H_4_	30
**13f**	H	Ph	38
**13g**	H	4-MeC_6_H_4_	32

## Data Availability

Data are contained within the article and Supplementary Materials.

## Supplementary Materials

The following supporting information can be downloaded at: https://www.mdpi.com/article/10.3390/molecules29174273/s1, Figure S1: Packing of molecules of **3c** looking down a-axis; Table S1: Cell viability of cancer cell lines after treatment with tetrahydroquinolines; Figure S2: ^1^H NMR of **8a**; Figure S3: ^13^C NMR of **8a**; Figure S4: ^1^H NMR of **8f**; Figure S5: ^13^C NMR spectrum of **8f**; Figure S6: ^1^H NMR of **8g**; Figure S7: ^13^C NMR spectrum of **8g**; Figure S8: ^1^H NMR of **10b**; Figure S9: ^13^C NMR spectrum of **10b**; Figure S10: ^1^H NMR of **10c**; Figure S11: NOESY NMR of **10c** showing interaction between H3 and H4; Figure S12: ^13^C NMR spectrum of **10c**; Figure S13: ^1^H NMR of **10g**; Figure S14: ^13^C NMR spectrum of **10g**; Figure S15: ^1^H NMR of **13f**; Figure S16: ^13^C NMR spectrum of **13f**.
