# Supplementary material for: Synthesis and Anticancer Activity of 3,4-Diaryl-1,2-dihydro- and 1,2,3,4-Tetrahydroquinolines
Source: Molecules. 2024 Sep 9;29(17):4273. doi: 10.3390/molecules29174273 (PMC11396787; doi:10.3390/molecules29174273)
Supplement: Supplementary file 1 [file molecules-29-04273-s001.zip › molecules-3116450-supplementary.pdf]

molecules

Supplementary information

Santosh Rajput [1], Valerio Falasca [1], Mohan Bhadbhade [2], David StC Black [1,*] and Naresh Kumar [1,*]

[1] School of Chemistry, University of New South Wales, Sydney, NSW 2052, Australia
[2] Solid State and Elemental Analysis Unit, Mark Wainwright Analytical Centre, The University of New South Wales, Sydney, NSW 2052, Australia
* Correspondence: d.black@unsw.edu.au (D.S.B.); n.kumar@unsw.edu.au (N.K.)

1.1 X-ray data for compound 3c

CCDC: 957124

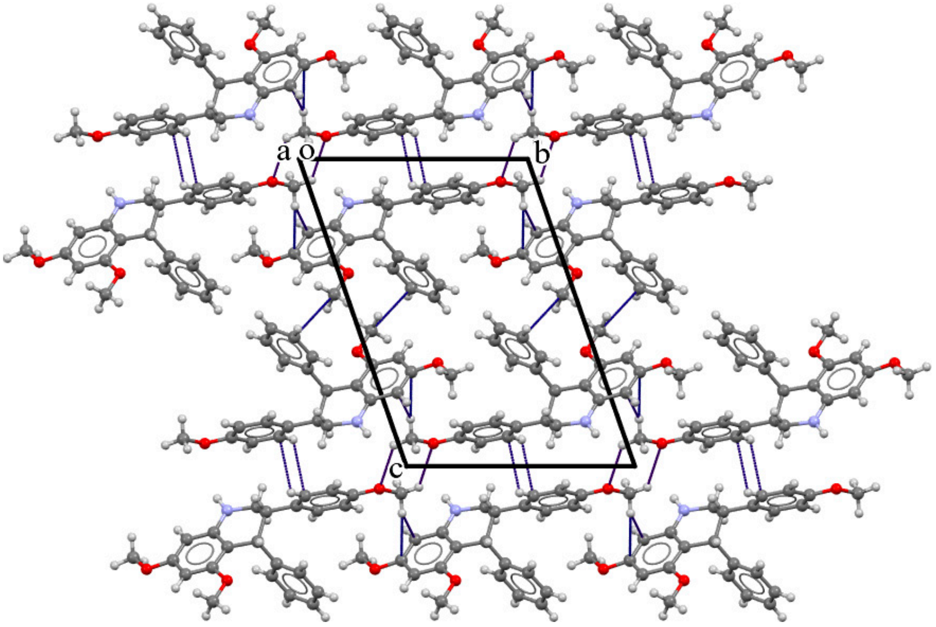

Figure S1: Packing of molecules of **3c** looking down a-axis

Packing of molecules is dominated by CH_3-π and slipped π-π interactions as shown in the Figure 1. The presence of three aromatic rings in the molecule essentially involves their association with weaker interactions of the said types.

1.2 Biological data

Table S1. Cell viability of cancer cell lines after treatment with tetrahydroquinolines.

Entry	H460	DU145	A-431	HT-29	MCF-7
**4b**	94.3 ± 4.2	91.1 ± 0.9	89.6 ± 1.8	105.7 ± 1.0	85.1 ± 4.8
**3b**	30.7 ± 0.8	104.5 ± 1.1	62.0 ± 1.6	63.4 ± 2.5	25.4 ± 1.6
**3c**	2.6 ± 0.1	11.9 ± 0.5	1.1 ± 0.2	3.3 ± 0.4	4.9 ± 0.2
**3e**	32.5 ± 0.3	100.2 ± 2.5	63.6 ± 1.0	50.1 ± 1.3	23.9 ± 1.5

1.3 NMR Spectra

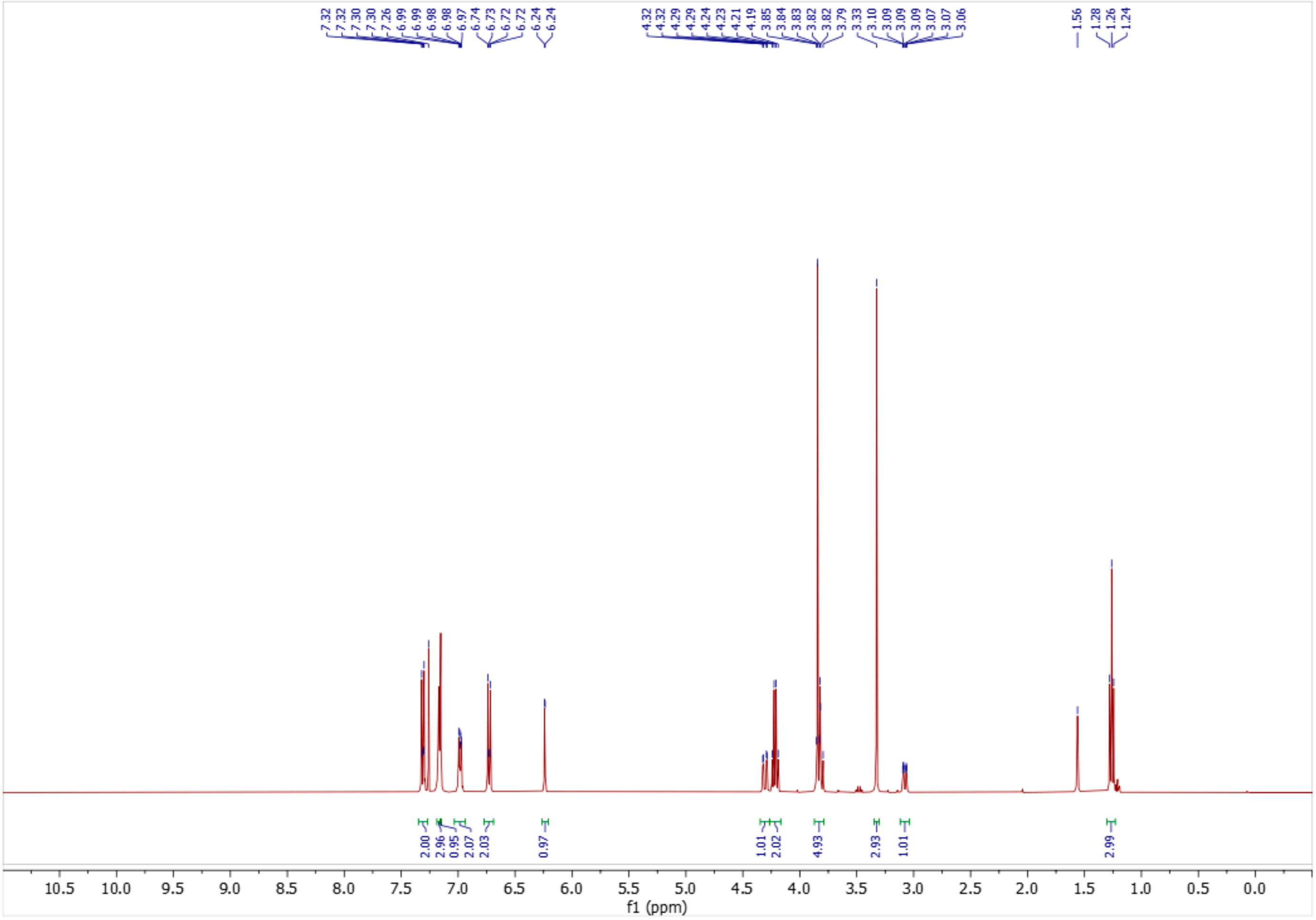

Figure S2. ^{1}H NMR of **8a**

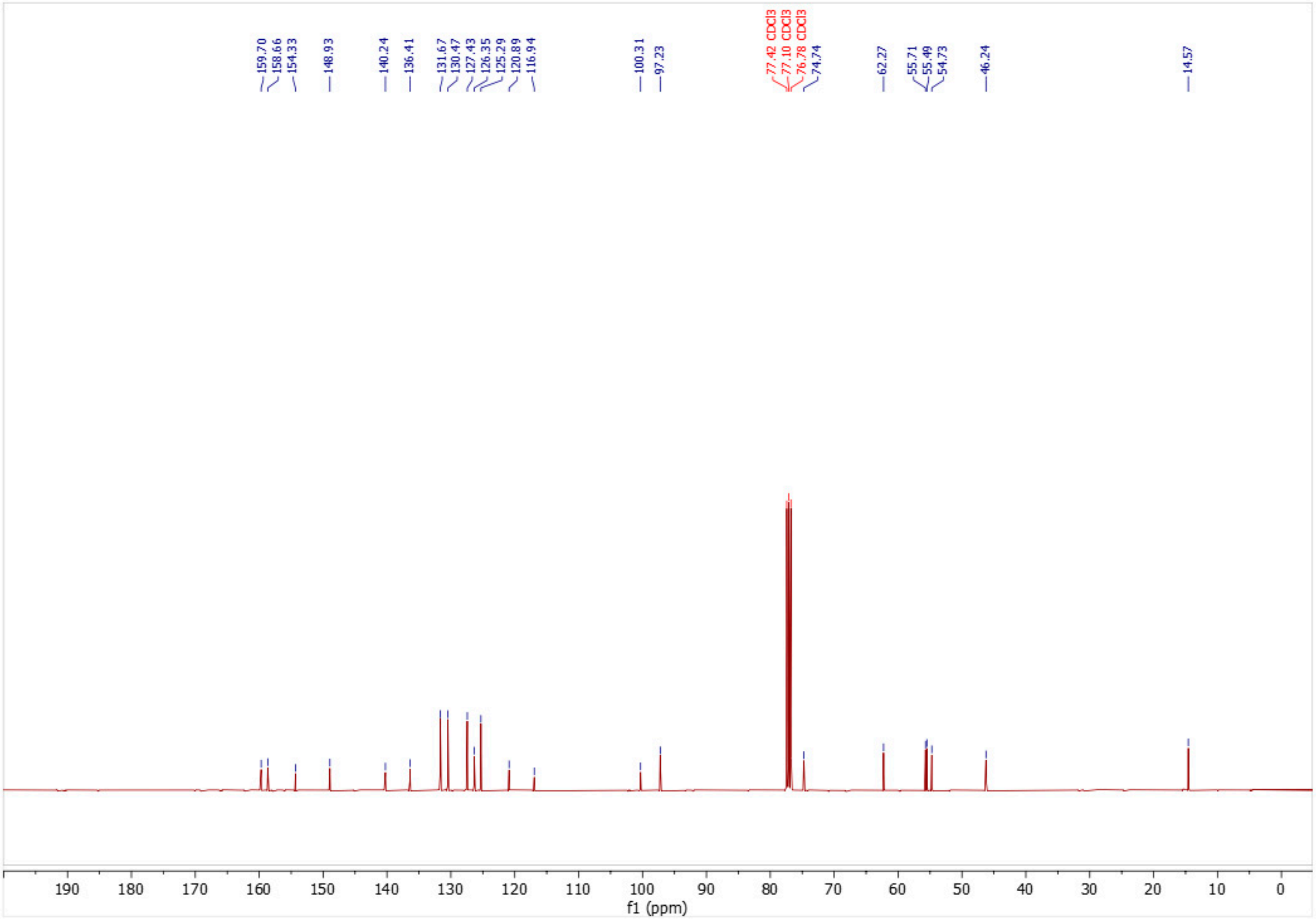

Figure S3. ^{13}C NMR of **8a**

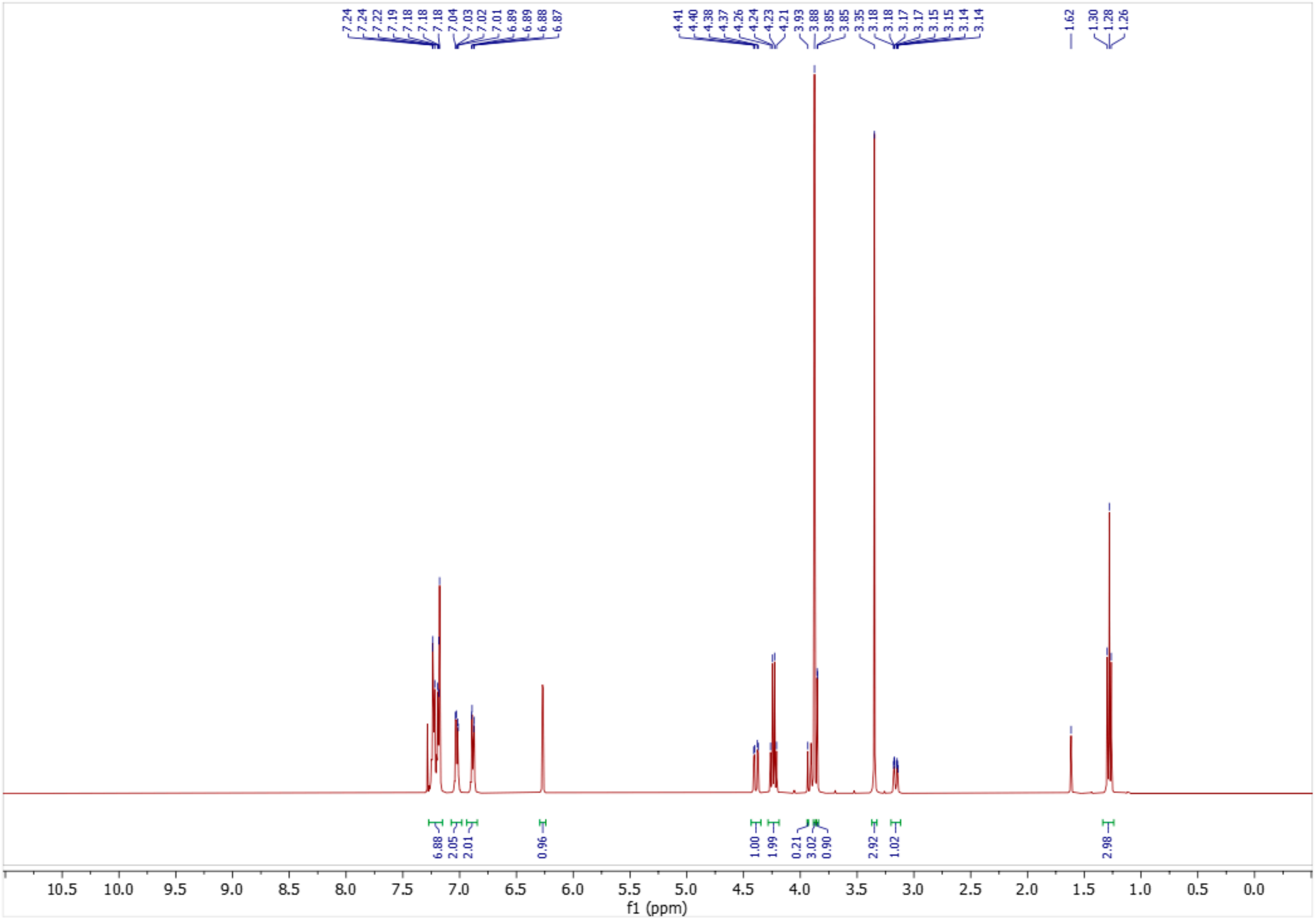

Figure S4. ^{1}H NMR of **8f**

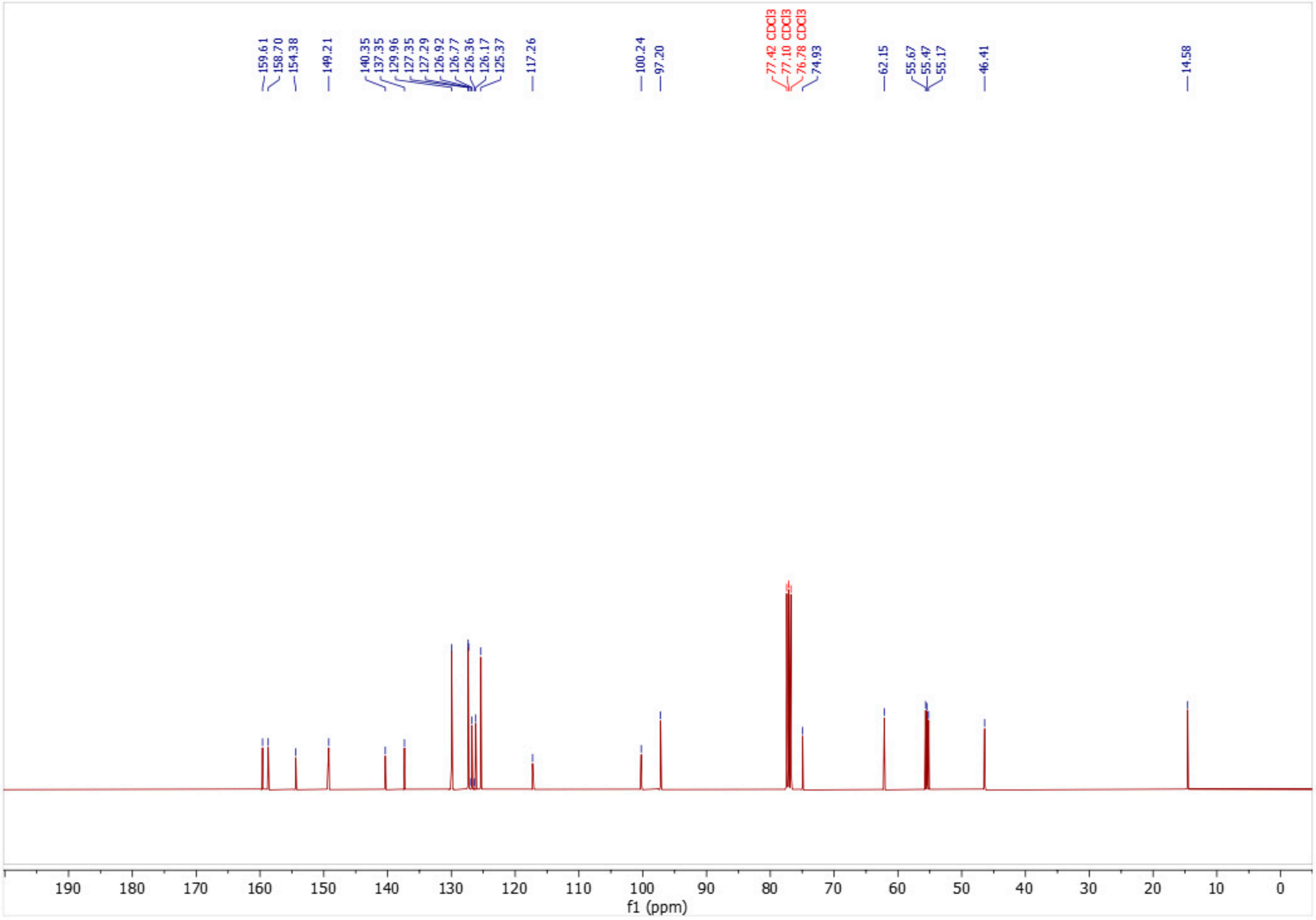

Figure S5. ^{13}C NMR spectrum of **8f**

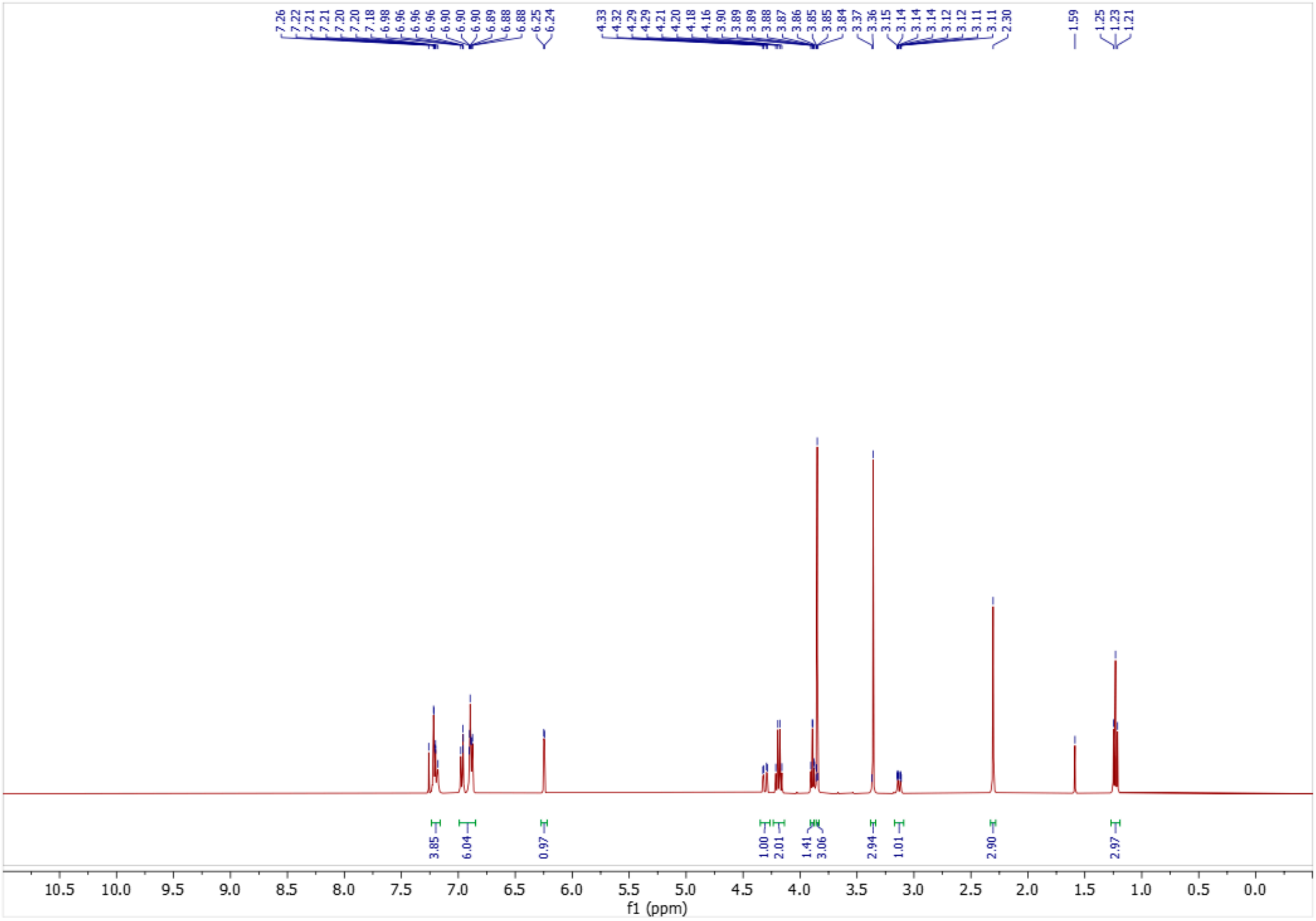

Figure S6. ^{1}H NMR of **8g**

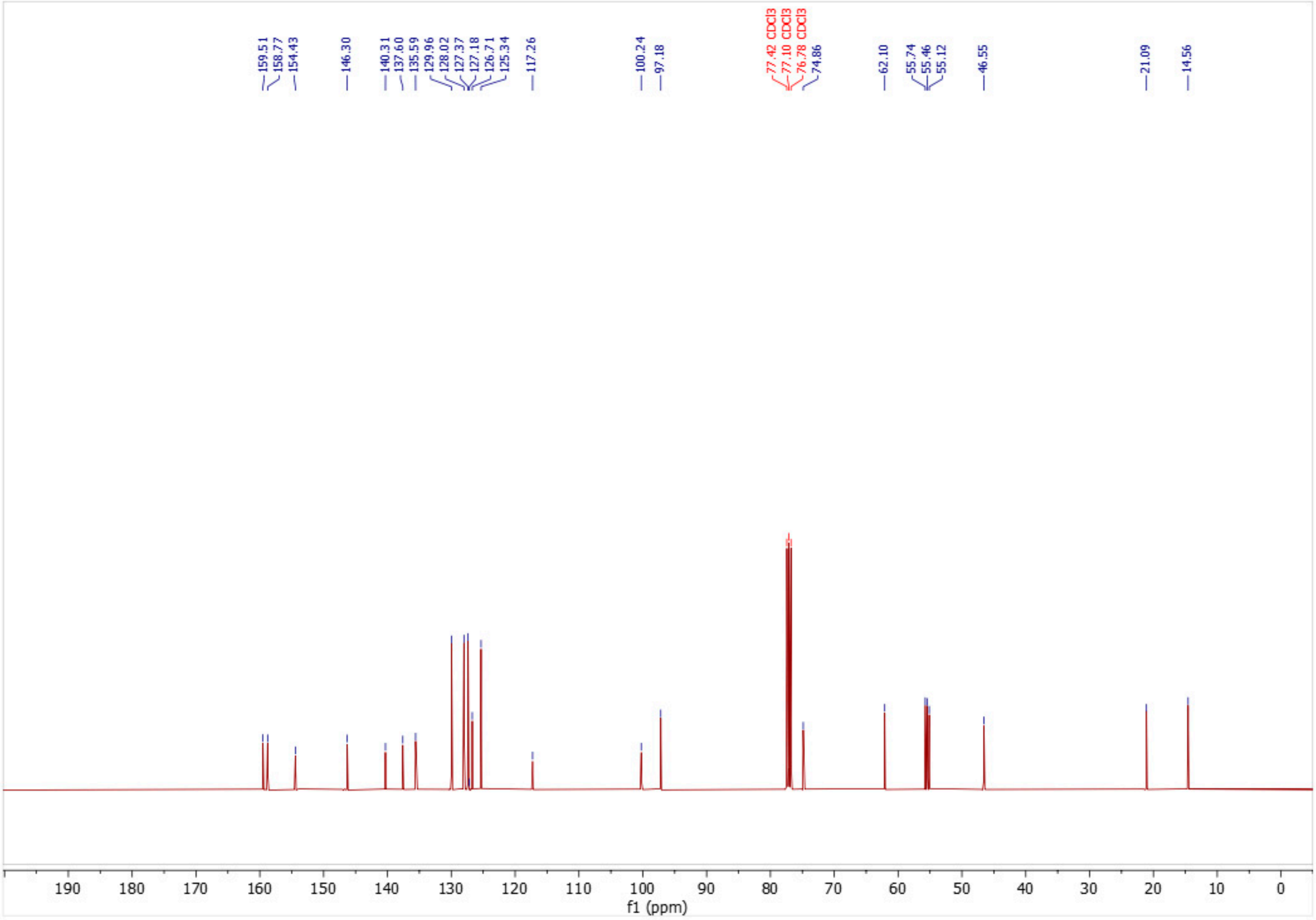

Figure S7. ^{13}C NMR spectrum of **8g**

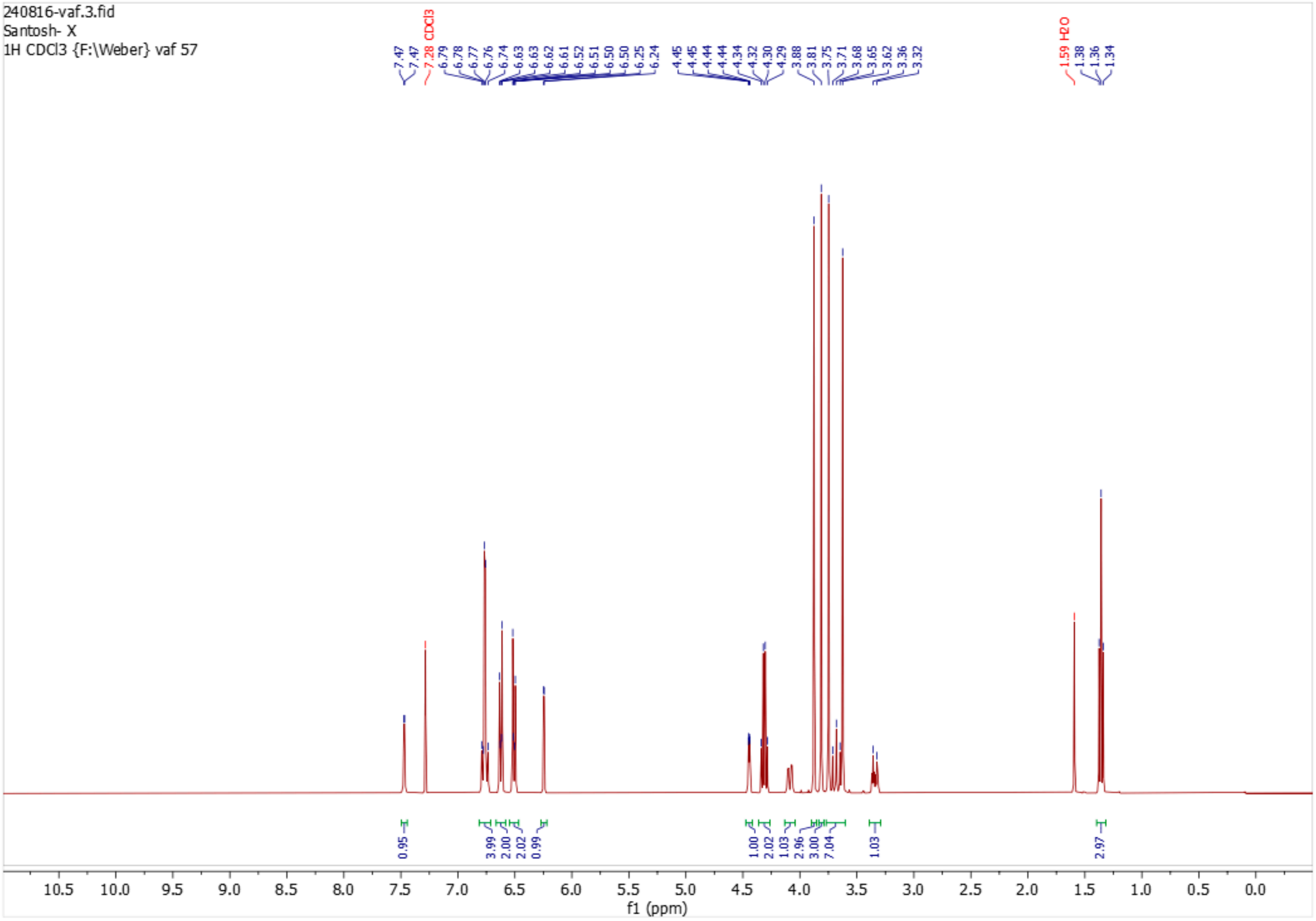

Figure S8. ^{1}H NMR of **10b**

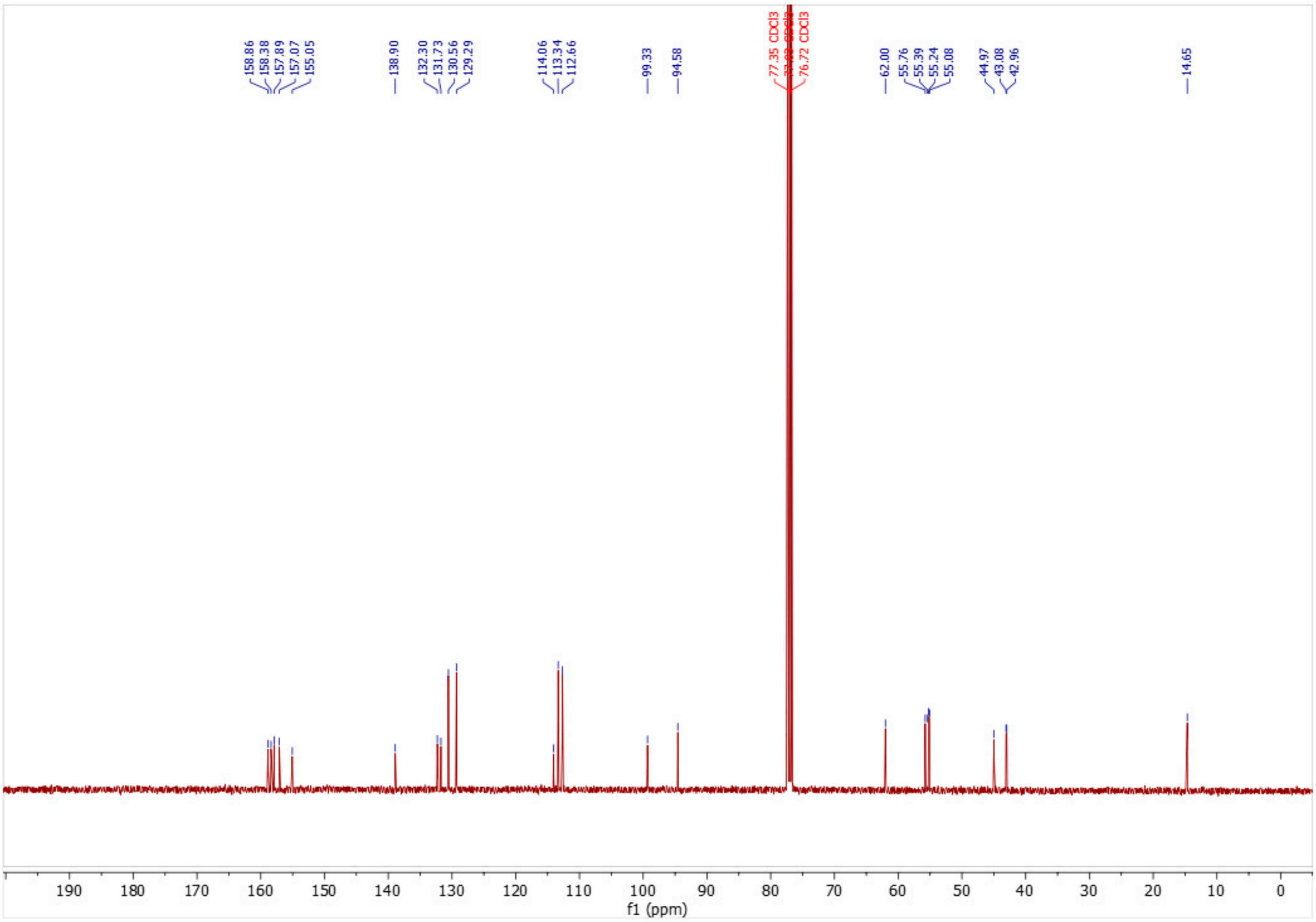

Figure S9. ^{13}C NMR spectrum of **10b**

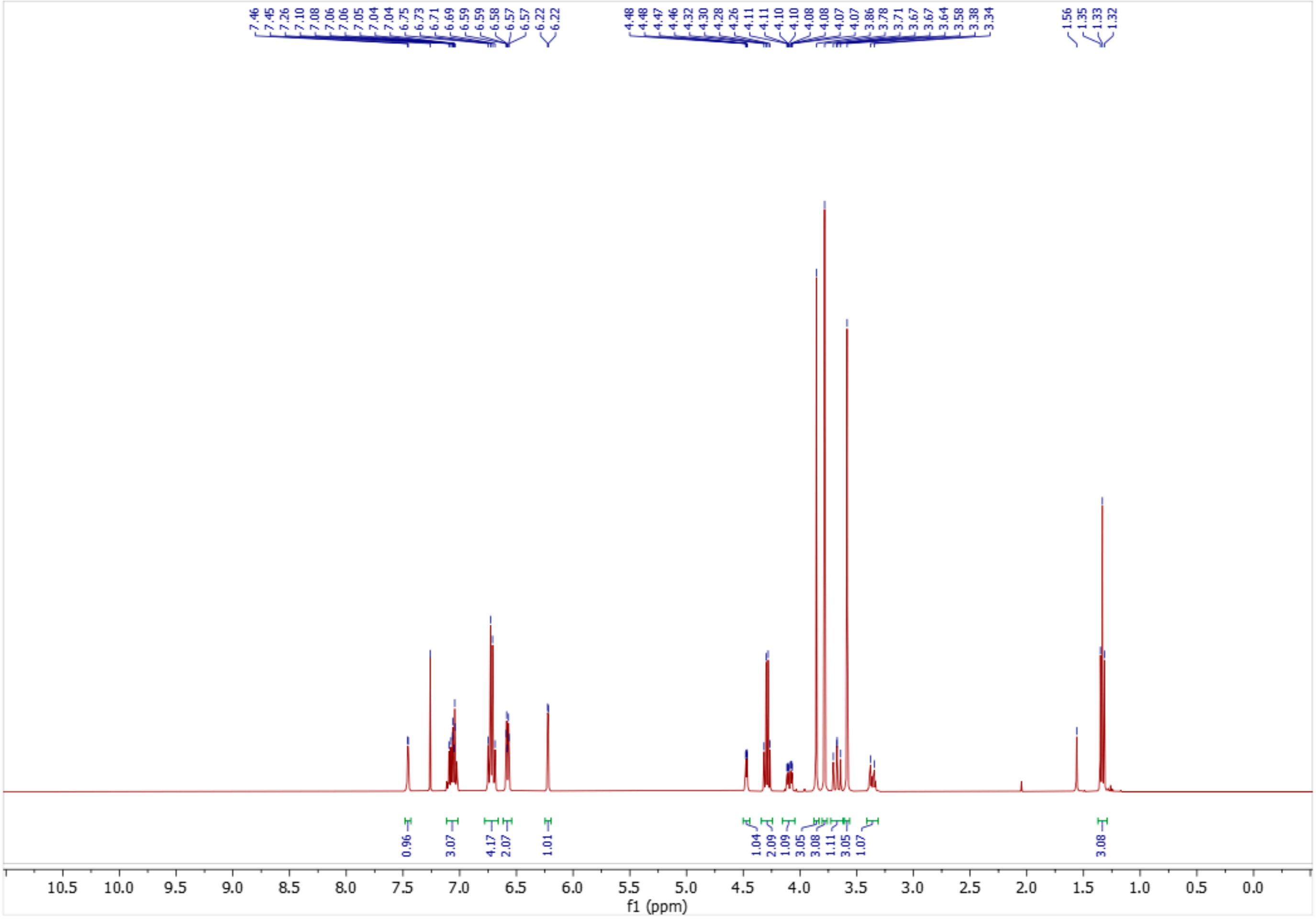

Figure S10. ^{1}H NMR of **10c**

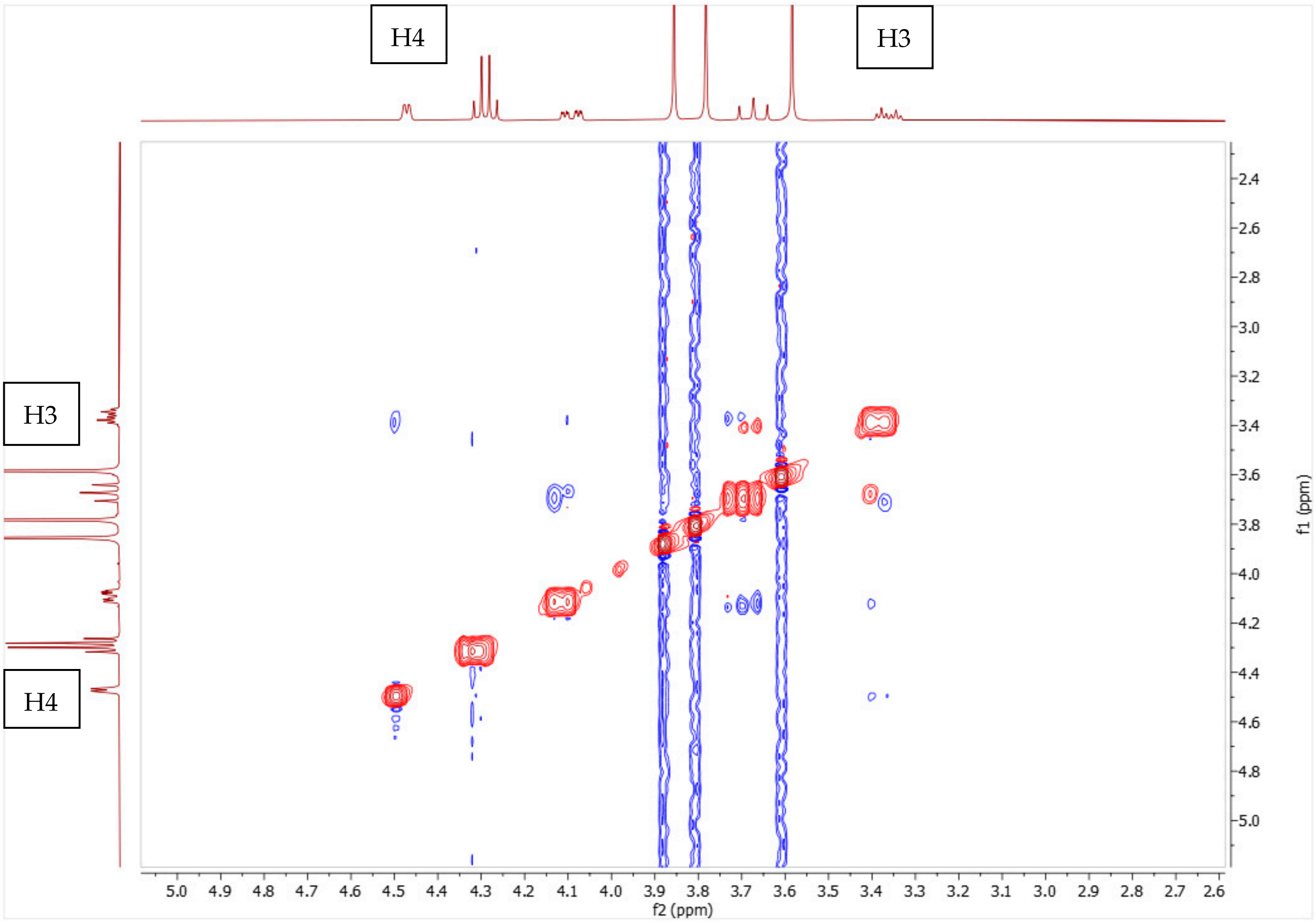

Figure S11. NOESY NMR of **10c** showing interaction between H3 and H4

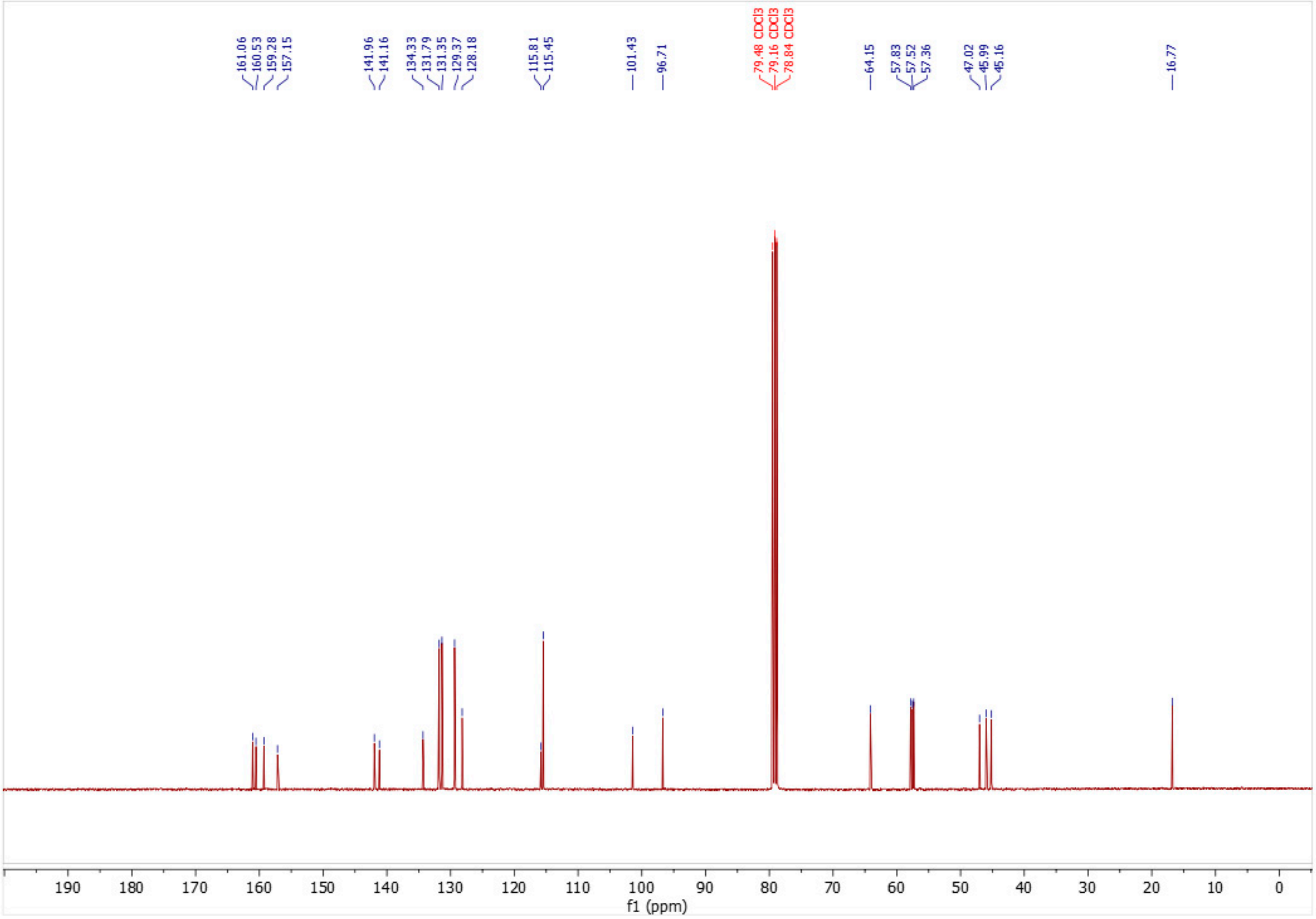

Figure S12. ^{13}C NMR spectrum of **10c**

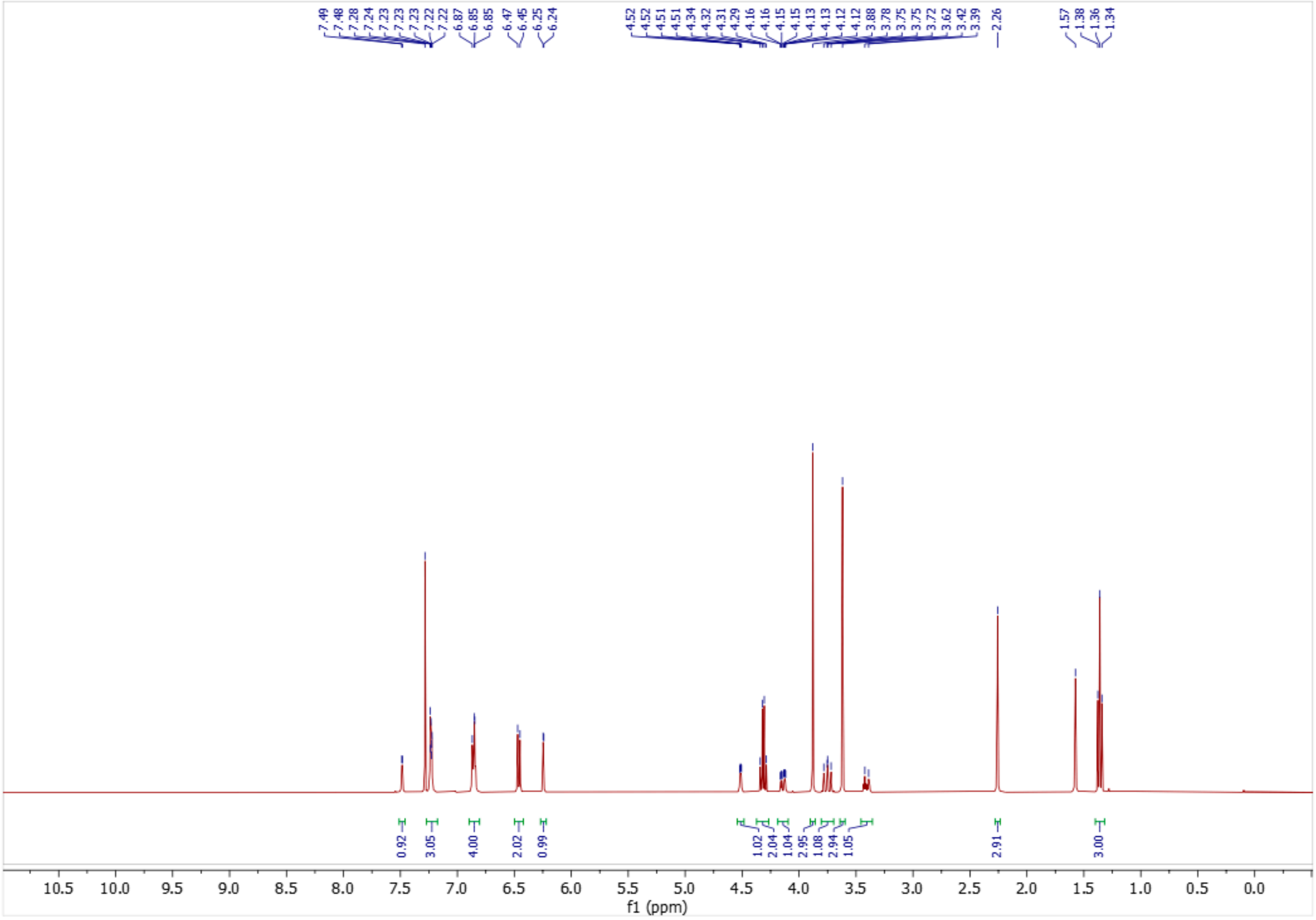

Figure S13. ^{1}H NMR of **10g**

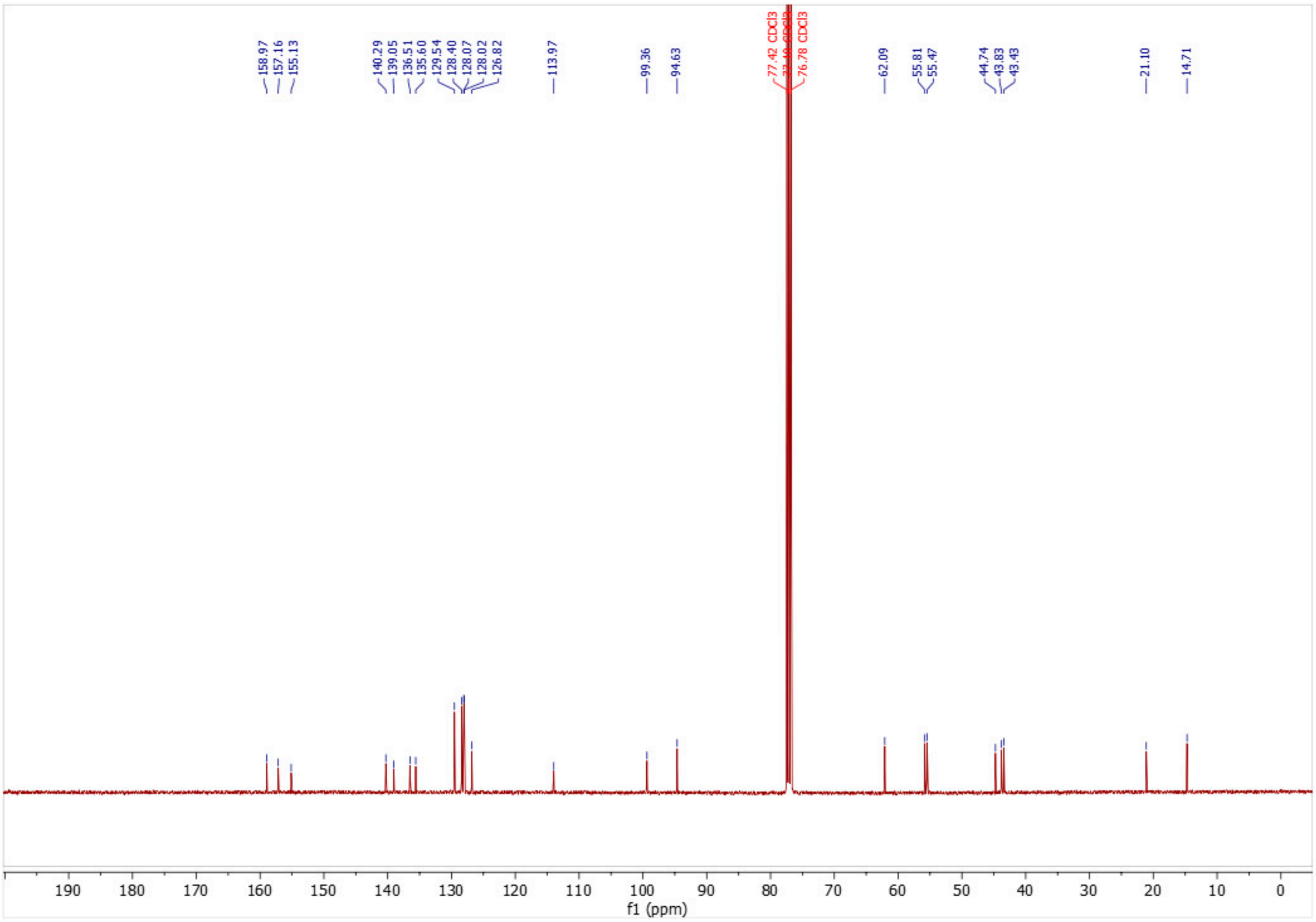

Figure S14. ^{13}C NMR spectrum of **10g**

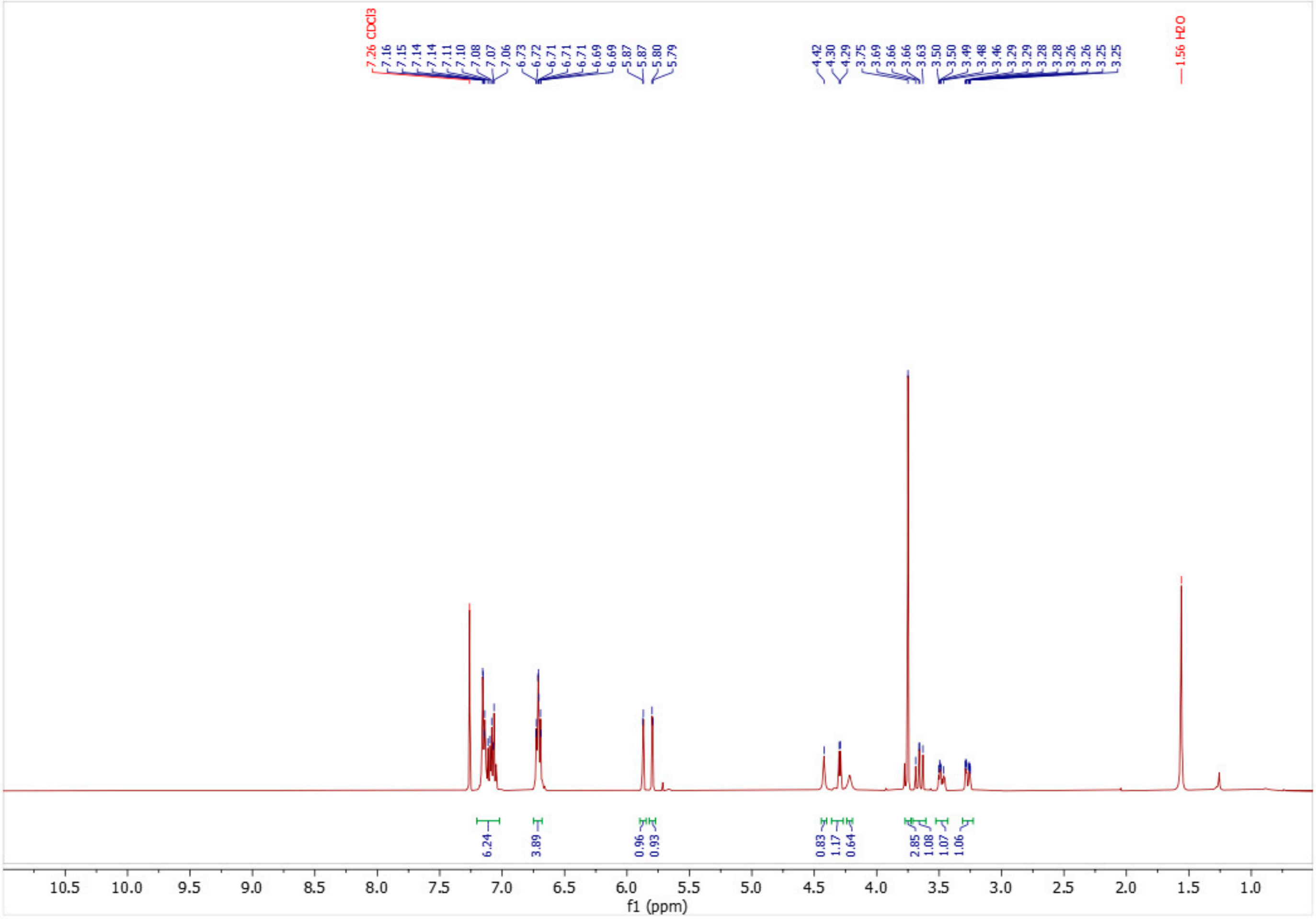

Figure S15. ^{1}H NMR of **13f**

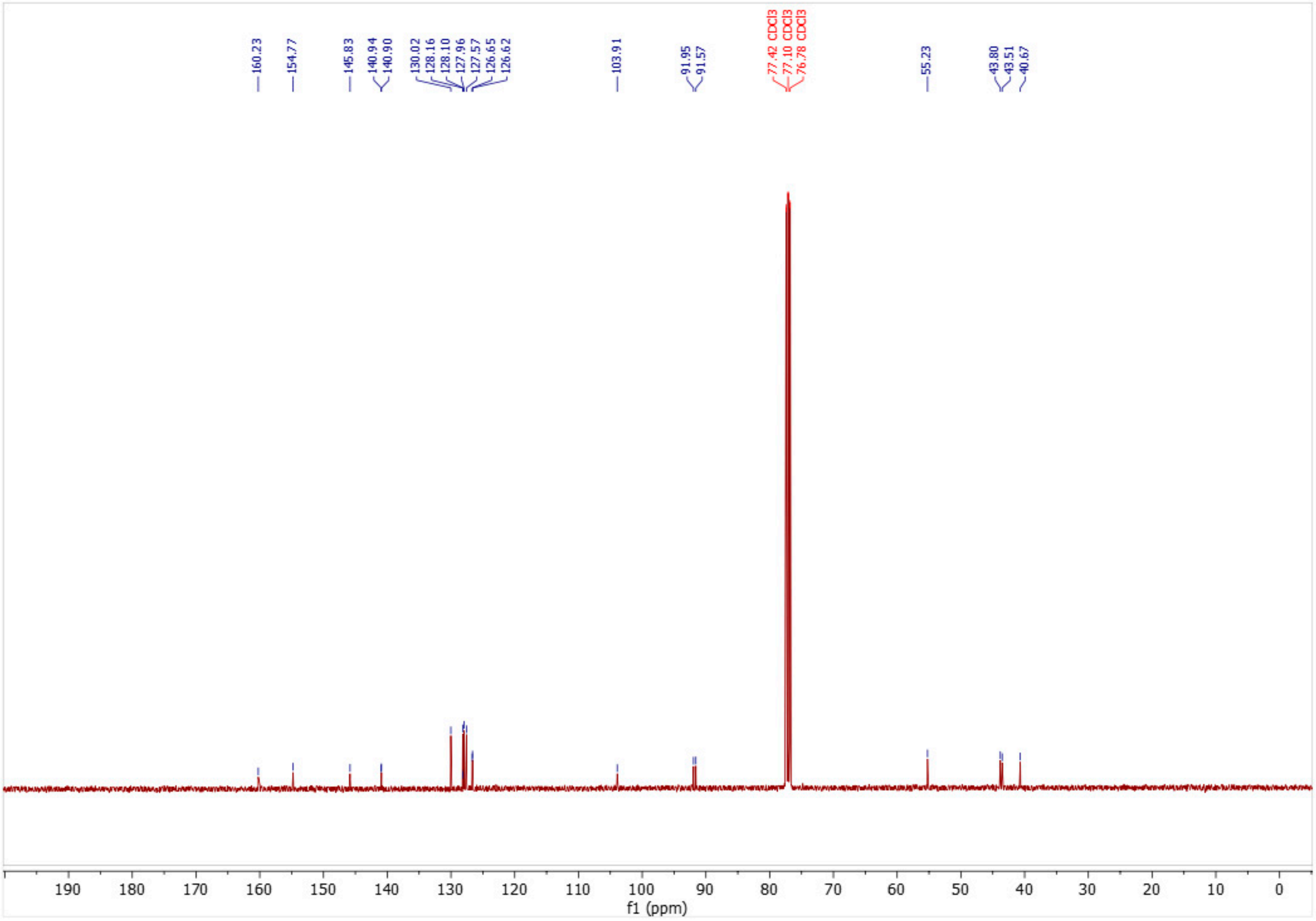

Figure S16. ^{13}C NMR spectrum of **13f**
